# Variations in the *FRA10AC1* Fragile Site and 15q21 Are Associated with Cerebrospinal Fluid Aβ_1-42_ Level

**DOI:** 10.1371/journal.pone.0134000

**Published:** 2015-08-07

**Authors:** Qingqin S. Li, Antonio R. Parrado, Mahesh N. Samtani, Vaibhav A. Narayan, Alzheimer’s Disease Neuroimaging Initiative

**Affiliations:** 1 Neuroscience Therapeutic Area, Janssen Research & Development, LLC, 1125 Trenton-Harbourton Road, Titusville, NJ 08560, United States of America; 2 Discovery Science, Janssen Research & Development, LLC, 1400 McKean Road, Spring House, PA 19002, United States of America; 3 Clinical Pharmacology, Advanced PK/PD Modeling and Simulation, Janssen Research & Development, LLC, 920 Route 202, Raritan, NJ 08869, United States of America; The University of Chicago, UNITED STATES

## Abstract

Proteolytic fragments of amyloid and post-translational modification of tau species in Cerebrospinal fluid (CSF) as well as cerebral amyloid deposition are important biomarkers for Alzheimer’s Disease. We conducted genome-wide association study to identify genetic factors influencing CSF biomarker level, cerebral amyloid deposition, and disease progression. The genome-wide association study was performed via a meta-analysis of two non-overlapping discovery sample sets to identify genetic variants other than *APOE* ε4 predictive of the CSF biomarker level (Aβ_1–42_, t-Tau, p-Tau_181P_, t-Tau:Aβ_1–42_ ratio, and p-Tau_181P_:Aβ_1–42_ ratio) in patients enrolled in the Alzheimer’s Disease Neuroimaging Initiative (ADNI) study. Loci passing a genome-wide significance threshold of *P* < 5 x 10^−8^ were followed-up for replication in an independent sample set. We also performed joint meta-analysis of both discovery sample sets together with the replication sample set. In the discovery phase, we identified variants in *FRA10AC1* associated with CSF Aβ_1–42_ level passing the genome-wide significance threshold (directly genotyped SNV rs10509663 *P*_FE_ = 1.1 x 10^−9^, imputed SNV rs116953792 *P*_FE_ = 3.5 x 10^−10^), rs116953792 (*P*_one-sided_ = 0.04) achieved replication. This association became stronger in the joint meta-analysis (directly genotyped SNV rs10509663 *P*_FE_ = 1.7 x 10^−9^, imputed SNV rs116953792 *P*_FE_ = 7.6 x 10^−11^). Additionally, we identified locus 15q21 (imputed SNV rs1503351 *P*_FE_ = 4.0 x 10^−8^) associated with CSF Aβ_1–42_ level. No other variants passed the genome-wide significance threshold for other CSF biomarkers in either the discovery sample sets or joint analysis. Gene set enrichment analyses suggested that targeted genes mediated by miR-33, miR-146, and miR-193 were enriched in various GWAS analyses. This finding is particularly important because CSF biomarkers confer disease susceptibility and may be predictive of the likelihood of disease progression in Alzheimer’s Disease.

## Introduction

Alzheimer’s Disease (AD) is the most common form of dementia and to date there is still no cure. Understanding the factors influencing cognitive decline of AD will enable us to better search for therapeutics to intervene or preempt this process. It is well established now that the CSF biosignature of increased total tau (t-tau) and phosphor-tau (p-tau) species especially tau phosphorylated at the threonine 181 (p-tau_181p_) and decreased amyloid-β 1–42 peptide (Aβ_1–42_), is predictive of Alzheimer’s related amyloid pathology in the brain.[1–4] Using the Alzheimer’s Disease Neuroimaging Initiative (ADNI) samples from ADNI-1 sample set, the APOE *ε4* allele best discriminated AD from controls (CN) in a logistic regression model including Aβ_1–42_, t-tau, while the AD-like t-tau/Aβ_1–42_ profile was detected among mild cognitive impairment (MCI) patients who converted to AD in one year follow up.[5] Samtani et al., used the ADNI-1 data to modeled disease progression using the Alzheimer’s Disease Assessment Scale-Cognitive Subscale (ADAS-Cog) in MCI patients using a mixture model and proposed log CSF p-tau_181p_:Aβ_1–42_ ratio > -1.86 to be the predictor discriminant progressers from non-progressers.[6] Other CSF biomarkers such as Aβ_1–42_, p-tau_181p_, and t-tau have also been linked to disease progression as measured by conversion from MCI to AD and/or cognitive decline. For example, the combination of CSF Aβ_1–42_ (or Aβ_1-42_/p-tau_181p_) and t-tau predicted the conversion from MCI to AD.[7] In a small study, non-demented patients with severely impaired episodic memory (SIM) but with no moderate impairment (MIM) or no impairment (NIM) at baseline declined cognitively over time and progressed to dementia at a high rate, and this was accompanied by significant increase in CSF p-tau_181P_ but not t-tau or Aβ_1–42_ during approximately 3 year follow-up.[8]

Most genetic studies in AD thus far have been utilizing clinical diagnosis to define cases and controls. These studies are inherently limited by accuracy of clinical diagnosis. For example, we know that up to 36.1% of APOE *ε4* allele non-carriers clinically diagnosed with Alzheimer’s Dementia do not have Alzheimer’s pathology as measured by PIB-PET.[9] GWAS studies that utilize PET amyloid signal or a CSF biosignture as quantitative endophenotype offer an opportunity, in principle, to define more objective phenotype, and establish direct associations between genetic variations, disease state biomarkers and disease progression. Kim et al. performed a GWAS to identify genetic risk factors for the three singular CSF biomarkers (Aβ_1–42_, p-tau_181p_, and t-tau) and two ratios (p-tau_181p_:Aβ_1–42_ and t-tau:Aβ_1–42_), utilized the ADNI1 samples genotyped with the Illumina Human 610-Quad BeadChip [10], they implicated one novel gene, *EPC2* that reached genome-wide significance for association with t-tau while confirming the expected association of CSF biomarkers with the *APOE/TOMM40* region. Cruchaga et al. identified a SNP located in intron 5 of the regulatory subunit of the *PPP3R1* gene associated with CSF p-tau_181_ levels in two independent CSF sample sets.[11] The largest CSF biomarker GWAS (N = 1,269) was based on a one stage analysis of four datasets leading to the identification of three novel genome-wide significant loci, an intronic imputed SNP in *GLIS3* (associated with p-tau_181p_ and t-tau), an intergenic imputed SNP between *GMNC* and *OSTN* (associate with t-tau), and an intergenic genotyped SNP (associated with p-tau_181p_) near *NCR2* and within the *TREM* gene cluster.[12] Thus far, the ADNI-GO/-2 CSF samples have not been included in any CSF GWAS analysis. In this study, we expanded the reported studies by including additional samples genotyped by the ADNI study and thus aimed to identify genetic variants predictive of CSF biomarkers independent of the *APOE* ε4 effect.

Similar to CSF Aβ_1–42_, cerebral amyloid deposition as measured by PET imaging has been used as a quantitative trait (QT) in GWAS, in addition to the *APOE* loci, rs509208 near butyrylcholinesterase (*BCHE*) was identified as a hit passing the genome-wide significant threshold for association with the florbetapir PET QT.[13] Hu et al., assessed the rate of disease progression in MCI subjects, as measured by changes in the Clinical Dementia Rating-sum of boxes (CDR-SB), by performing a GWAS in two independent sample sets and identified several novel loci that achieved genome-wide significance (intronic SNPs in *UBR5* and *PARP6*, and an intergenic SNP near *ACOT11*).[14] In this report, we also describe GWAS or GWAS meta-analysis using florbetapir PET as a quantitative trait and using a dichotomized measure of amyloid positivity, and disease progression/rate of cognitive decline in late mild cognitive impairment (LMCI) population.

## Results

**Fig 1:**
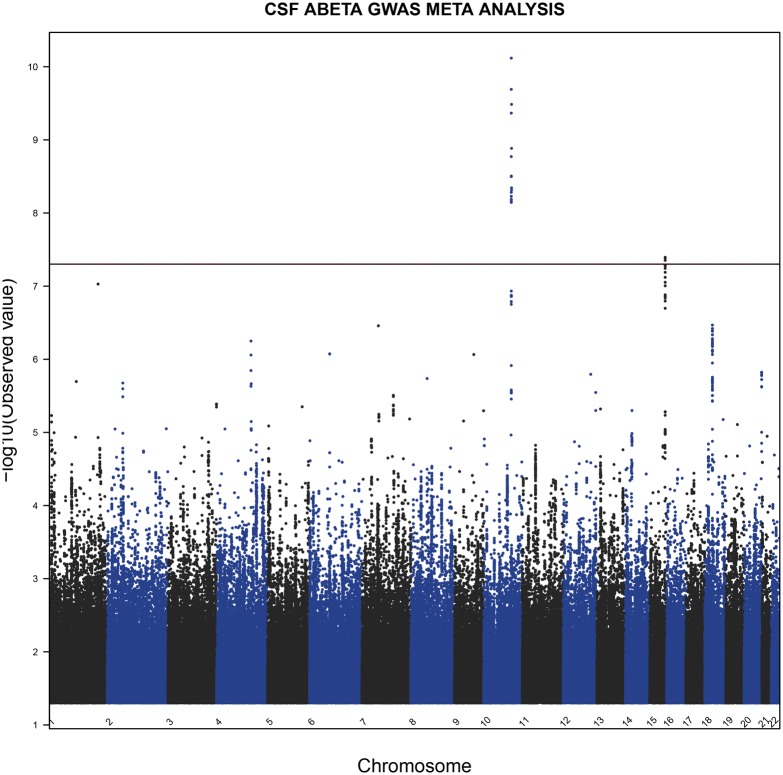
Manhattan plot of the CSF Biomarker Aβ_1–42_ GWAS Meta-Analysis. The dotted line indicates genome wide significance threshold of 5x10^-8^. Only variants with p < 0.05 are shown.

**Fig 2:**
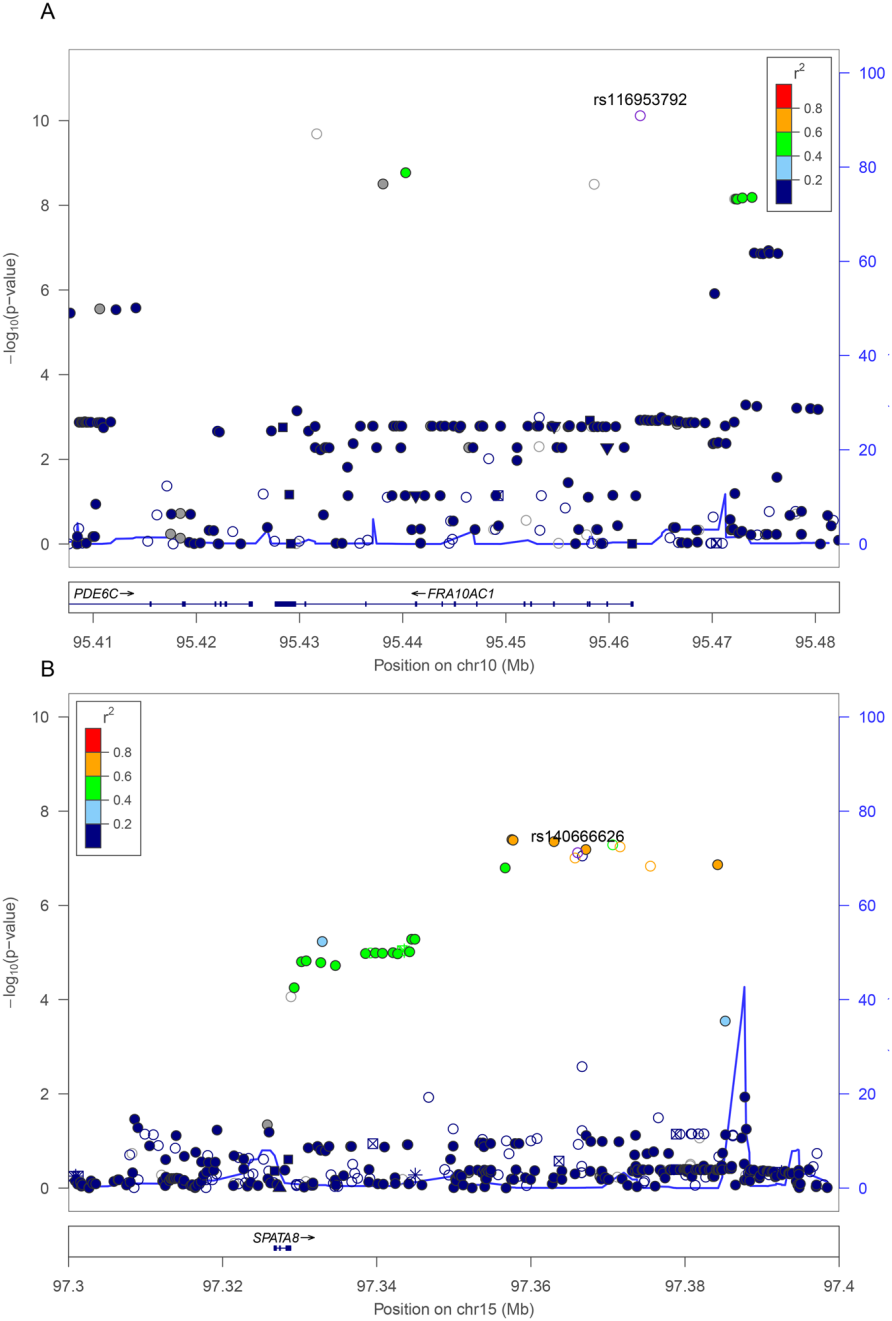
Regional Plot for the CSF Aβ_1–42_ Loci. **(A) *FRA10AC1* (B) 15q21** Association results (-log10 p) are plotted for all single nucleotide polymorphisms (SNPs) passing quality control. Chromosome position is plotted with reference to the NCBI build 37. Recombination rate as estimated from the HapMap Project is plotted in light blue. SNPs are color coded according to the LD measure (r^2^) with reference SNP based on the reference panel of CEU population from the 1000 Genome Project (March 2012 release). SNP annotation for all 1000GP SNPs are represented by the annotation categories: framestop (triangle), splice (triangle), non-synonymous (inverted triangle), synonymous (square), UTR (square), TFBScons (star), MCS44 Placental (square with diagonal lines) and none-of-the-above (filled circle).

**Fig 3:**
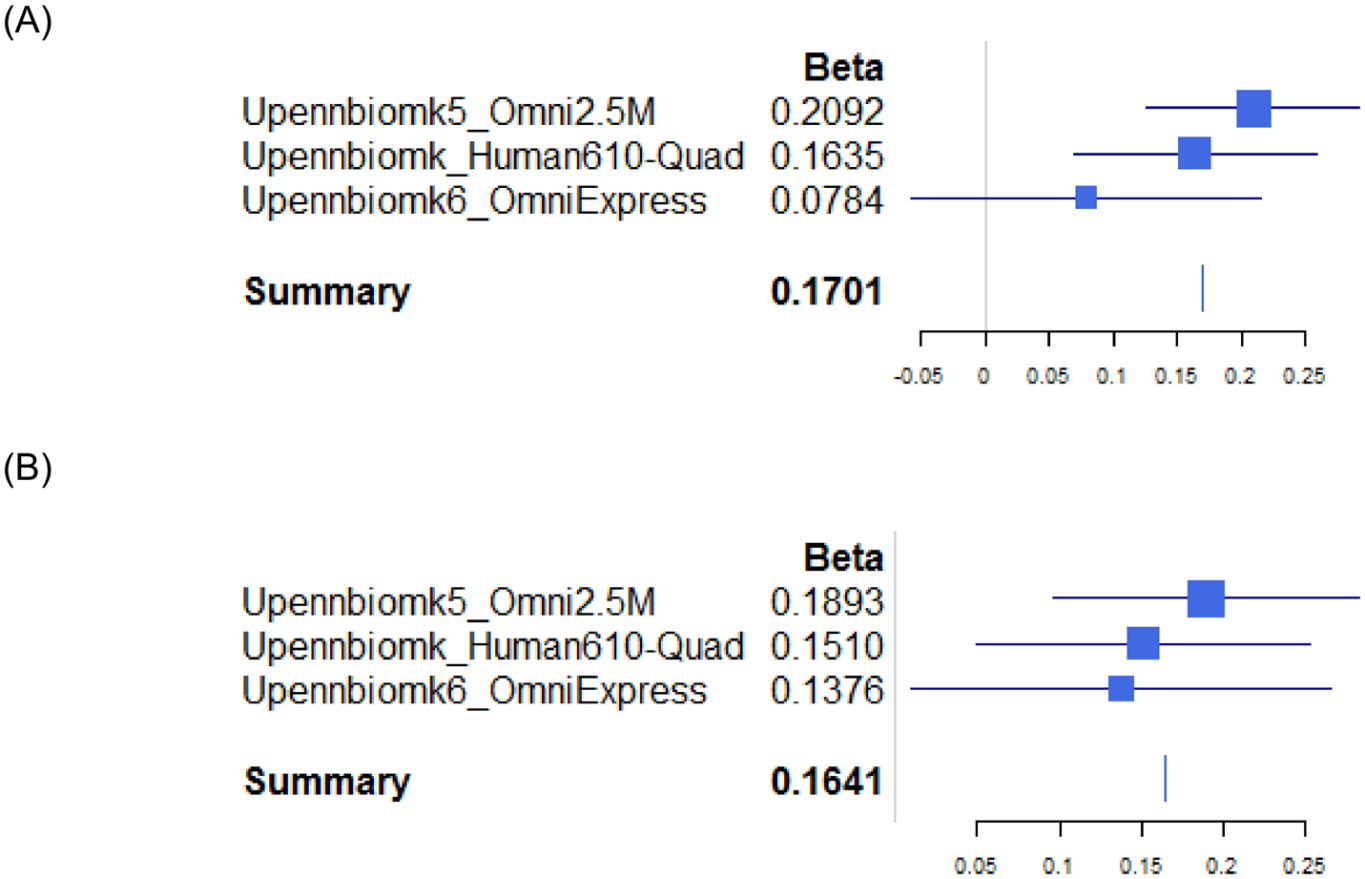
Forest Plot for the CSF Aβ_1–42_ Loci. (A) rs10509663 in *FRA10AC1* (B) rs4301994 in 15q21 showing the effect of genotype on Aβ_1–42_ by Individual sample set.

We performed a genome-wide association study via a meta-analysis of two discovery sample sets (discovery sample set 1: genotyped using the Illumina Human610-Quad; discovery sample set 2: genotyped using Illumina Omni2.5, Table 1 and Table A in S1 File) to identify genetic variants other than *APOE* ε4 predictive of the CSF biomarker level (Aβ_1–42_, t-Tau, p-Tau_181P_, t-Tau:Aβ_1–42_ ratio, and p-Tau_181P_:Aβ_1–42_ ratio) in patients enrolled in the ADNI study, and followed-up by replication of any locus passing the genome-wide significance threshold of *P* < 5 x 10^−8^. In the discovery phase, we identified variants from one single locus associated with CSF Aβ_1–42_ level passing the genome-wide significance threshold. The most significantly associated markers predictive of CSF Aβ_1–42_ level in our meta-analysis of discovery sample sets are, directly genotyped intronic SNV rs10509663 *P*_FE_ = 1.1 x 10^−9^ and imputed putative promoter region SNV rs116953792 *P*_FE_ = 3.5 x 10^−10^, they are located in a ~60kb interval in chromosome 10 that contains the rare FRA10A folate-sensitive fragile site *FRA10AC1* (Figure B1 in S1 File). This genetic association was replicated (rs116953792 *P*_one-sided_ = 0.04) in the replication sample set (Table 1 and Table A in S1 File, n = 172 genotyped using Illumina OmniExpress). We also performed joint meta-analysis of both discovery sample sets together with the replication sample set. The *FRA10AC1* association became stronger in the joint analysis (directly genotyped SNV rs10509663 *P*_FE_ = 1.7 x 10^−9^, imputed SNV rs116953792 *P*_FE_ = 7.6 x 10^−11^, Figs 1 and 2A). We identified an additional genome wide-significant locus within the15q21 locus (directly genotyped SNV rs4301994 *P*_FE_ = 6.5 x 10^−8^; imputed SNV rs1503351 *P*_FE_ = 4.0 x 10^−8^, Figs 1 and 2B) also associated with CSF Aβ_1–42_ level. This locus is in the intergenic region of chromosome 15 (15q21) between spermatogenesis associated 8 (*SPATA8*) and hypothetical *LOC91948*. There are spliced yet uncharacterized EST in this “intergenic” region. No other variants passed the genome-wide significance threshold level for any of the other CSF biomarkers (t-Tau, p-Tau_181P_, t-Tau:Aβ_1–42_ ratio, and p-Tau_181P_:Aβ_1–42_ ratio, Figures B2-5 in S1 File). As expected, *APOE* ε4, age, and clinical diagnosis were predictive of CSF Aβ_1–42_ level in all three sample sets. The association between *FRA10AC1* locus and CSF Aβ_1–42_ is primarily driven by the first two sample sets with uncorrected significance levels of p = 0.0006 for the Upennbiomk_Human610-Quad sample set and p = 1.86 x 10^−6^ for the Upennbiomk5_Omni2.5 sample set, and *P*_two-sided_ = 0.2 (*P*_one-sided_ = 0.1) for the Upennbiomk6_OmniExpress sample set for the directly genotyped marker rs10509663. The imputed SNV rs116953792 however achieved *P*_one-sided_ = 0.04 for the Upennbiomk6_OmniExpress sample set. The association between rs4301994 and CSF Aβ_1–42_ is supported by all three sample sets with uncorrected significance levels of *P* = 0.003 for the Upennbiomk_Human610-Quad sample set, *P* = 6.6 x 10^−5^ for the Upennbiomk5_Omni2.5 sample set, and *P* = 0.03 in the Upennbiomk6_OmniExpress sample set. The regression beta coefficients and least square means of the minor allele dosage for rs10509663 (*FRA10AC1*) and rs4301994 (15q21 locus) are shown in the forest plot (Fig 3A and 3B) and Table 2, displaying a consistent trend across the three sample sets. Both directly genotyped markers rs10509663 and rs4301994 did not deviate from Hardy-Weinberg Equilibrium (*P* = 0.38 and 1, respectively based on Omni2.5 data among cognitively normal controls). Table 3 contains those mostly independent variants (both the most significant directly genotyped and imputed markers are retained) that are significantly associated with Aβ_1–42_ level from our meta-analysis, along with other variants with uncorrected *P* ≤ 1x10^-6^ in any CSF biomarker meta-analysis. The full list of variants meeting this more liberal threshold appears in the S1 Table.

**Table 1:** Basic demographic information of the three ADNI CSF Sample Sets.

	Upennbiomk_Human610-Quad (N = 391)	Upennbiomk5_Omni2.5 (N = 385)	Upennbiomk6_OmniExpress (N = 204)
Sex, n (%)			
F	155 (39.6)	176 (45.7)	87 (42.6)
M	236 (60.4)	209 (54.3)	117 (57.4)
Age at baseline, years			
Mean (SD)	74..8 (7.1)	72.5 (7.5)	72.1 (7.6)
Median (Range)	75 (54.4, 89.6)	72.6 (55.0, 91.4)	72.3 (48.1, 89.3)
Baseline Clinical Diagnosis, n (%)			
CN	109 (27.9)	106 (27.5)	21 (10.3)
SMC	N/A	N/A	3 (1.5)
EMCI	N/A	189 (49.1)	57 (27.9)
LMCI	186 (47.6)	65 (16.9)	61 (29.9)
AD	96 (24.6)	25 (6.5)	62 (30.4)
APOE ε4, copy, n (%)			
0	198 (50.6)	234 (60.9)	76 (39.8)
1	149 (38.1)	122 (31.8)	82 (42.9)
2	44 (11.3)	28 (7.3)	33 (17.3)
Missing Data	0	1	13
CDR-SB 0 n (%)	103 (26.3)	100 (26.0)	24 (11.8)
Aβ_1–42_ (pg/ml)			
Mean (SD)	170.2 (56.6)	176.6 (50.3)	160.4 (51.7)
Median (Range)	152.5 (53, 300)	175.5 (82.5, 313.6)	146.3 (40.5, 301.9)
p-Tau_181P_ (pg/ml)			
Mean (SD)	33.8 (18.5)	39.6 (23.2)	44.2 (25.9)
Median (Range)	30 (8, 115)	33.7 (9.4. 173.3)	38.9 (6.9, 151.2)
t-tau (pg/ml)			
Mean (SD)	98.4 (55.8)	80.3 (47.4)	106.8 (64.1)
Median (Range)	84 (28, 495)	66 (19.9, 300.5)	89.1 (23.2, 360.0)

**Table 2:** Summary Characteristics of top CSF Aβ1–42 variants by Sample Set averaging over the levels of APOE ε4 and baseline clinical diagnosis assuming the baseline age was 60.

Sample Set	Upennbiomk_Human610-Quad		Upennbiomk5_Omni2.5		Upennbiomk6_OmniExpress	
Copy	N	Least square means ± standard error (SE)	N	Least square means ± standard error (SE)	N	Least square means ± standard error (SE)
rs10509663-G						
0	313	161.8 ± 5.8	313	171.2 ± 4.9	154	159.6 ± 6.3
1	24	146.7 ± 10.6	30	141.6 ± 8.2	16	149.8 ± 11.7
2	3	96.3 ± 27.1	1	62.9 ± 39.5	-	-
rs4301994-C						
0	311	162.5 ± 5.9	316	169.9 ± 4.9	154	159.9 ± 6.0
1	28	141.1 ± 10.1	28	139.6 ± 8.6	18	139.2 ± 11.2
2	1	151.3 ± 46.5	-	-	-	-

**Table 3:** Summary of CSF biomarker GWAS meta-analyses—SNPs with uncorrected p-value less than 1x10^-6^. Top variants were clumped using parameters—clump-p1 0.000001—clump-p2 0.05—clump-r2 0.2—clump-range entrez.gene.map—clump-range-border 20.

SNP^a^	CHR	BP^b^	A_1_	A_2_	Func	Gene	MAF^c^	Imputed_2.5M/610K/OmniExpress_	P_Discovery_ ^d^	P_replication, 1-sided_ ^d^	P_joint_ ^d^	β_joint_ ^e^
*A*β_*1–42*_												
rs116953792	10	95463026	G	T	upstream	**FRA10AC1**	0.01	Y/Y/Y	3.47E-10	0.039115	**7.64E-11**	0.2395
rs10509663	10	95440286	A	G	intronic	**FRA10AC1**	0.14	N/N/N	1.08E-09	0.12455	**1.69E-09**	0.1701
rs1503351	15	97357520	A	G	intergenic	**SPATA8(dist = 28675)**,**LOC91948(dist = 928326)**	0.05	Y/Y/Y			**4.03E-08**	0.1699
rs4301994	15	97367115	T	C	intergenic	SPATA8(dist = 38270),LOC91948(dist = 918731)	0.06	N/N/N			6.47E-08	0.1641
rs188308056	15	97366666	T	A	intergenic	SPATA8(dist = 37821),LOC91948(dist = 919180)	0.03	Y/Y/Y			8.88E-08	0.3122
rs75849835	1	221475899	A	G	intergenic	HLX(dist = 417499),C1orf140(dist = 27371)	0.02	Y/Y/Y			9.38E-08	0.2042
rs7098209	10	95475470	T	C	intergenic	FRA10AC1(dist = 13141),LGI1(dist = 42096)	0.25	N/N/N			1.17E-07	0.0885
rs509477	18	32559295	C	G	intronic	MAPRE2	0.6	Y/Y/Y			3.41E-07	-0.0653
rs55704525	7	43568566	G	A	intronic	HECW1	0.01	Y/Y/Y			3.48E-07	0.3463
rs140913323	4	125740709	T	G	intergenic	ANKRD50(dist = 106822),FAT4(dist = 496858)	0.02	Y/Y/Y			5.64E-07	0.4822
rs2493168	6	53118235	A	G	intergenic	GCM1(dist = 104611),ELOVL5(dist = 13961)	0.71	Y/Y/Y			8.39E-07	-0.0653
rs8190569	9	98998061	T	G	intronic	HSD17B3	0.01	Y/Y/Y			8.61E-07	0.24
*p-Tau*_*181P*_												
rs6005807	22	28934313	T	C	intronic	TTC28	0.9	Y/Y/Y			2.74E-07	-0.213
rs75213930	1	83125608	G	A	intergenic	LPHN2(dist = 667501),MIR548AP(dist = 1133990)	0.03	Y/Y/Y			3.59E-07	0.299
rs76478271	19	41325199	T	A	intergenic	RAB4B-EGLN2(dist = 10853),CYP2A6(dist = 24244)	0.12	Y/Y/Y			4.08E-07	-0.3309
*t-Tau*												
rs76137255	19	40783832	T	G	intronic	AKT2	0.02	Y/Y/Y			2.45E-07	-0.3236
rs79811809	7	140633481	A	G	intergenic	BRAF(dist = 8917),MRPS33(dist = 72480)	0.04	Y/Y/Y			3.31E-07	0.2394
rs36056951	8	139965798	C	T	intergenic	COL22A1(dist = 39562),KCNK9(dist = 659006)	0.03	Y/Y/Y			3.88E-07	-0.3291
rs138451097	19	2873629	A	G	intronic	ZNF556	0.01	Y/Y/Y			5.39E-07	-1.0045
rs8045334	16	63573376	G	A	intergenic	CDH8(dist = 1502637),CDH11(dist = 1407307)	0.19	N/N/N			8.15E-07	-0.126
*p-Tau*_*181P*_:*Aβ*_*1–42*_ *ratio*												
rs2301659	19	19035354	G	T	intronic	DDX49	0.32	Y/Y/Y			2.66E-07	-0.1679
rs117025875	8	6999135	G	A	intergenic	DEFA5(dist = 84876),LOC349196(dist = 119006)	0.02	Y/Y/Y			9.33E-07	-0.8594
*t-Tau*:*Aβ*_*1–42*_ *ratio*												
rs113027826	2	207549512	T	C	intronic	DYTN	0.14	Y/Y/Y			3.71E-07	-0.4164
rs114162361	1	174575642	C	T	intronic	RABGAP1L	0.01	Y/Y/Y			9.39E-07	-0.6189

Chr, chromosome; A_1_, first allele code; A_2_, second allele code

^a^ Indexed SNPs with uncorrected p < 1x10^-6^ in any of the CSF Biomarker GWAS Meta-Analyses

^b^ Build 37, assembly hg19

^c^ based on 2012 Apr release of 1000genome and all population

^d^ Fixed-effects meta-analysis p-value

^e^ Beta coefficient of for the SNP assuming additive genetic model

Although the CSF Aβ_1–42_ measurement using Upennbiomk5 (baseline Aβ_1–42_ for 117 ADNI-GO subjects and 272 ADNI-2 subjects) were not taken at the exact same visit as the florbetapir PET imaging, the overall correlation for the overlapping subjects between the two measurements was negatively correlated (r = -0.72). We expected the results from the cerebral amyloid deposition GWAS to provide complementary evidence with CSF Aβ_1–42_ meta-analysis even if the sample sets are not identical and the endpoints are not perfectly negatively correlated. In our florbetapir PET GWAS meta-analysis without correcting for *APOE* ε4 dosage, the *APOE* locus predicted florbetapir PET SUVR value (directly genotyped SNV rs429358 *P* = 7.99 x 10^−32^) as expected from the published results (Figure C1 in S1 File). There are uncommon variants (such as rs76117213, an intronic variant in WD repeat and FYVE domain containing 3 (*WDFY3*), *P* = 1.39 x 10^−7^ without correction for *APOE* ε4 dosage, *P* = 4.08 x 10^−6^ with correction for *APOE* ε4 dosage). The uncommon variant results, however, shall be interpreted with caution as these variants occurred at low minor allele frequency and the association statistics are based on small sample size for rare genotype groups. Table 4 contains the variants that were significant in florbetapir PET GWAS along with other variants with uncorrected *P* < 1x10^-6^ in any florbetapir PET GWAS analysis, most variants are independent except for the chromosome 19 region. The full list of variants meeting this more liberal threshold appears in the S2 Table. Top 15q21 locus variants associated with CSF Aβ_1–42_ exhibited a nominal association with florbetapir PET SUVR value (*P* = 0.002 for directly genotyped SNV rs4301994; *P* = 0.001 for imputed SNV rs1503351). Similarly, the imputed variant rs116953792 from *FRA10AC1* also exhibited a nominal association with florbetapir PET SUVR value (*P* = 0.01). Results for the top CSF Aβ_1–42_ variant rs10509663 from *FRA10AC1* in this and other analyses (S3 Table) are discussed in the S1 File.

**Table 4:** Summary of cerebral amyloid deposition florbetapir PET quantitative traits—SNPs with uncorrected p-value less than 1x10^-6^. Top variants were clumped using parameters—clump-p1 0.000001—clump-p2 0.05—clump-r2 0.2—clump-range entrez.gene.map—clump-range-border 20.

SNP^a^	CHR	BP^b^	Func	Gene	MAF^c^	Imputed_2.5M/0mni Express_	A_1_	A_2_	P^d^	β^e^	ExonicFunc
*AV45 (not correcting APOE ε4)*											
rs76117213	4	85596236	intronic	WDFY3	0.01	Y/Y	G	A	1.39E-07	-0.26	
rs362902	6	146681157	intronic	GRM1	0.01	Y/Y	T	C	6.30E-07	-0.27	
rs36116061	8	89373327	intergenic	MMP16(d ist = 33610), RIPK2(dist = 1396648)	0.18	N/Y	T	G	6.93E-07	0.06	
rs708886	12	119958702	intronic	CCDC60	0.44	Y/Y	T	G	8.47E-07	-0.05	
rs28810	16	3500515	intronic	NAA60	0.12	Y/Y	A	G	4.78E-07	0.06	
rs589398	18	5311892	intergenic	ZBTB14(dist = 14840),EPB41 L3(dist = 80496)	0.99	Y/Y	C	T	3.48E-07	0.19	
rs7407664	18	9031719	intergenic	SOGA2(d ist = 198944), NDUFV2(dist = 70909)	0.03	Y/Y	A	G	3.01 E-07	-0.14	
rs6859	19	45382034	UTR3	PVRL2	0.61	N/N	A	G	1.68E-07	0.05	
rs157580	19	45395266	intronic	T0MM40	0.63	N/N	G	A	1.63E-07	-0.05	
rs429358	19	45411941	exonic	**APOE**	0.15	Y/Y	T	C	**7.99E-32**	-0.13	nonsynonymous SNV
rs1081105	19	45412955	dow nstream	**APOE**	0.02	Y/Y	A	C	**1.48E-08**	-0.13	
rs12721052	19	45421972	intronic	AP0C1	0.2568	Y/Y	AG	A	1.65E-07	0.06	
rs60049679	19	45429708	upstream	**APOC1P1**	0.11	Y/Y	G	C	**1.30E-08**	-0.14	
*AV45 (correcting APOE ε4)*											
rs200527573	1	212742242	intronic	ATF3	0.0042	Y/Y	C	CTATT	7.90E-07	-0.28	
rs57450513	5	141225446	intergenic	ARAP3(dist = 163646), PCDH1 (dist = 7227)	0.01	Y/Y	C	A	2.42E-07	-0.22	
rs708886	12	119958702	intronic	CCDC60	0.44	Y/Y	T	G	9.33E-07	-0.05	
rs28810	16	3500515	intronic	NAA60	0.12	Y/Y	A	G	5.90E-07	0.06	

Chr, chromosome; A_1_, first allele code; A_2_, second allele code

^a^ Representative SNPs with uncorrected p < 1x10^-6^ in any of the Cerebral amyloid deposition GWAS Analyses

^b^ Build 37, assembly hg19

^c^ based on 2012 Apr release of 1000genome and all population

^d^ Fixed-effects meta-analysis p-value

^e^ Beta coefficient of for the SNP assuming additive genetic model

For the rate of cognitive decline GWAS in the late-MCI subgroup, there were some variants that achieved the conventional genome-wide significance threshold (Figure D in S1 File), those variants occurred at low frequency (MAF < 0.05). An intronic variant rs2694777 in the GDNF family receptor alpha 1 gene (*GFRA1*) is among the common variants with suggestive association with rate of cognitive decline as measured by rate of change in CDR-SB (*P* = 1.2 x 10^−5^ European ancestry sample set and *P* = 6.77 x 10^−6^ in all races). The full list of variants with *P* ≤ 10^−6^ appears in the S4 Table.

The results from this study, for variants reported in the literature relevant/associated with CSF biomarkers, florbetapir PET, and disease progression were also reported in the S1 File.

Gene set enrichment analysis may yield signals of enriched gene sets in GWAS analysis despite the individual variants not reaching genome wide significance. Applying INRICH [15] enrichment analysis to the CSF biomarker GWAS results yields gene sets such as miR-33 target genes (*P*_empirical_ = 0.0002, *P*_corrected_ = 0.03) being enriched among p-tau_181p_:Aβ1–42 suggestive association hits (*P* < 0.0005) (Table 5 and S5 Table). miR-33 was identified to be a potent post-transcriptional regulator of lipid metabolism genes [16, 17] and cause disruption of cellular cholesterol homeostasis leading to pathologic processes including AD. Among, the potential targets of miR-33, *PRKAA1* (Protein kinase, AMP-activated, alpha 1 catalytic subunit) mediates an autophagic process to clear extracellular Aβ fibrils by microglia, the immune cells in the brain.[18] Other potential targets of miR-33 included *ARID5B* (AT rich interactive domain 5B (MRF1-like)), *KCNMA1* (Potassium large conductance calcium-activated channel, subfamily M, alpha member 1), and *LGI1* (leucine-rich, glioma inactivated). *ARID5B* was previously implicated in AD. *ARID5B* variants (rs2588969 and rs494288) showed marginally significant association with LOAD in meta-analysis of 2,634 LOAD and 4,201 controls (*P* = 0.046 and 0.008, respectively), although the associations did not survive adjustment for covariates (*P* = 0.30 and 0.11, respectively).[19] Other inconclusive association of *ARID5B* variants included Naj et al. (OR = 0.93, *P* = 0.001) and Hollingworth et al. (OR = 1.06, *P* = 0.03) for rs2588969 with LOAD.[20, 21] rs16934131 in *KCNMA1* was significantly associated with age at onset (AAO) of LOAD (P = 0.0066) and disease duration (P = 0.0002). *LGI1* is an extracellular matrix (ECM) molecule forming a complex with postsynaptic scaffolding proteins (postsynaptic density proteins 95 and 93, and the synapse-associated protein 97), presynaptic scaffolding proteins (Ca2+/calmodulinactivated serine-threonine kinase and Lin7), and presynaptic K+ channels (K_v_1.1, K_v_1.4 and K_v_β1 subunits) and demonstrated to be important in epilepsy, but ECM and chondroitin sulfate proteoglycans (CSPGs), one of the most abundant glycanated protein types found in the nervous system and a major ECM component, which form dense lattice-like structures, termed perineuronal nets (PNNs), are thought to be neuroprotective in AD.[22] Application of Aβ_1–42_ to rodent primary neuronal cultures caused neuronal death of neurons not associated with PNNs, while the neurons expressing PNNs were not affected.[23] The same miR-33 target genes were observed to be enriched in other CSF biomarker suggestive hits, although the corrected enrichment p-value is greater than 0.05. In addition, miR-193 and miR-146 target genes are also enriched, miR-193 was one of the nine down-regulated miRNA identified in adult-onset AD Drosophila brains.[24] miR-193b were all upregulated in oxidative stressed (i.e. H_2_O_2_-induced) primary hippocampal neurons and different strains of senescence accelerated mice.[25]. miR-146 was reported to be related to up-regulated immune and inflammatory in Alzheimer's disease. [26]

**Table 5:** Inrich Analysis Results (P_corrected_ < 0.05).

Aβ_1–42_
CORTICAL_ACTIN_CYTOSKELETON http://www.broadinstitute.org/gsea/msigdb/cards/CORTICAL_ACTIN_CYTOSKELETON.html
GO:0005681 spliceosomal complex
p-tau_181p_
CLATHRIN_COATED_VESICLE http://www.broadinstitute.org/gsea/msigdb/cards/CLATHRIN_COATED_VESICLE.html
COATED_VESICLE http://www.broadinstitute.org/gsea/msigdb/cards/COATED_VESICLE.html
p-tau_181p_:Aβ_1–42_
05410 Hypertrophic_cardiomyopathy_(HCM)
CAATGCA,MIR-33 http://www.broadinstitute.org/gsea/msigdb/cards/CAATGCA,MIR-33.html
CELL_CELL_ADHESION http://www.broadinstitute.org/gsea/msigdb/cards/CELL_CELL_ADHESION.html
CELL_PROJECTION http://www.broadinstitute.org/gsea/msigdb/cards/CELL_PROJECTION.html
GNF2_PPP6C http://www.broadinstitute.org/gsea/msigdb/cards/GNF2_PPP6C.html
KEGG_HYPERTROPHIC_CARDIOMYOPATHY_HCM http://www.broadinstitute.org/gsea/msigdb/cards/KEGG_HYPERTROPHIC_CARDIOMYOPATHY_HCM.html
POSITIVE_REGULATION_OF_CELL_PROLIFERATION http://www.broadinstitute.org/gsea/msigdb/cards/POSITIVE_REGULATION_OF_CELL_PROLIFERATION.html
RIBONUCLEOPROTEIN_COMPLEX http://www.broadinstitute.org/gsea/msigdb/cards/RIBONUCLEOPROTEIN_COMPLEX.html
SPLICEOSOME http://www.broadinstitute.org/gsea/msigdb/cards/SPLICEOSOME.html
t-tau
CAGCACT,MIR-512-3P http://www.broadinstitute.org/gsea/msigdb/cards/CAGCACT,MIR-512-3P.html
AV45 QTL (correcting ε4)
GGCCAGT,MIR-193A,MIR-193B http://www.broadinstitute.org/gsea/msigdb/cards/GGCCAGT,MIR-193A,MIR-193B.html
PHOSPHORIC_ESTER_HYDROLASE_ACTIVITY http://www.broadinstitute.org/gsea/msigdb/cards/PHOSPHORIC_ESTER_HYDROLASE_ACTIVITY.html
AV45 QTL (not correcting ε4)
ACTIVATION_OF_JNK_ACTIVITY http://www.broadinstitute.org/gsea/msigdb/cards/ACTIVATION_OF_JNK_ACTIVITY.html
AGTTCTC,MIR-146A,MIR-146B http://www.broadinstitute.org/gsea/msigdb/cards/AGTTCTC,MIR-146A,MIR-146B.html
TGTGTGA,MIR-377 http://www.broadinstitute.org/gsea/msigdb/cards/TGTGTGA,MIR-377.html

## Discussion

In this study we have analyzed the ADNI Cohort to predict CSF and PET biomarker status and cognitive decline rates from genetic data and to assess if use of molecular biomarkers as quantitative traits provides extra power to uncover novel genotype-phenotype relationships in AD. Unequivocally, *APOE* ε4 allele is the strongest genetic predictor of CSF biomarker level, cerebral amyloid deposition, and disease progression. The effect size for other genetic markers is much smaller.

CSF biomarker measurements have the advantage that their measurements are widely sensitive across different patient subpopulations ranging from cognitive normal to AD. Cognitive measurements on the other hand are sensitive for different patient population segments and this will limit the sample size available for GWAS if we study the rate of cognitive decline directly in the selected patient population. For example, the Clinical Dementia Rating-Sum of Boxes (CDR-SB) is most sensitive for mild cognitive impairment (MCI) patients, while the Alzheimer’s Disease Assessment Scale-Cognitive Subscale (ADAS-Cog) will be more appropriate for AD patients. In this sample set, variants in *FRA10AC1* and 15q21 were associated with CSF Aβ_1–42_ reaching genome-wide significance, replication was achieved with the *FRA10AC1* variant. *FRA10AC1* (fragile site, folic acid type, rare, fra(10)(q23.3) or fra(10)(q24.2) candidate 1) encodes a nuclear phosphorprotein of unknown function. The 5' UTR of this gene is part of a CpG island and contains a tandem CGG repeat region that normally consists of 8–14 repeats but can expand to over 200 repeats. The CGG repeat is ~723 base pairs away from the rs116953792, the most significant SNP (*P* = 2.0 x 10^−10^, in LD with the directly genotyped variant rs10509663) associated with CSF Aβ_1–42_ level. The expanded allele becomes hypermethylated and is not transcribed and an expanded repeat region has not been associated with any disease phenotype. The extent of LD between the CpG repeat and rs116953792, is unclear, given their close proximity.

*WDFY3* is one of the biologically most interesting genes identified to have suggestive association with the florbetapir PET quantitative trait with or without correction for *APOE* ε4 dosage. *WDFY3* encodes a phosphatidylinositol 3-phosphate-binding protein that functions as a master conductor for aggregate clearance by autophagy. This protein shuttles from the nuclear membrane to co-localize with aggregated proteins, and complexes with other autophagic components to achieve macroautophagy-mediated clearance of aggregated proteins. This protein is of particular interest given the proposed synergy between amyloid and tau aggregates in driving AD progression. Another variant (rs17009220, *P* = 0.00484, http://www.gwascentral.org/marker/HGVM16286779/results?t=2) in *WDFY3* exhibited nominally significant interaction (SNP*APOE ε4) in an AD case-control GWAS study.[27]

*GFRA1* gene, which encodes GDNF family receptor alpha 1, a member of the GDNF receptor family, is among the few genes with suggestive association to rate of cognitive decline in the LMCI subgroup. The GDNF family receptor alpha 1 is a glycosylphosphatidylinositol(GPI)-linked cell surface receptor for both glial cell line-derived neurotrophic factor (*GDNF*) and neurturin (*NTN*), and mediates activation of the RET tyrosine kinase receptor. *GDNF* and *NTN* are two structurally related, potent neurotrophic factors that play key roles in the control of neuron survival and differentiation. Neuronal loss is a hallmark of AD, a neurodegenerative disease.

Human and mouse experiments have implicated the role of miRNA in the regulation of Aβ, tau, inflammation, and cell death as the main disease mechanisms of AD [28]. In our GWAS and INRICH analyses, it was intriguing that molecules involved in Aβ autophagy, inflammation, cell death and proliferation are enriched in INRICH analysis or among the top hits of GWAS analyses. *APOE* ε4 has been the most convincing genome wide signal in AD, and variants in *APOE-APOC1-APOC4-APOC2* and *TOMM40-APOE* have previously been associated with total cholesterol, LDL cholesterol, and triglyceride concentrations.[29, 30] Cholesterol metabolism was implicated to be enriched in the etiology of AD in previous study.[31] In the INRICH analysis, miR-33 targets are enriched in CSF GWAS analysis and miR-33 is critical in regulating cholesterol metabolism, affirming the interrelationship between cholesterol metabolism and AD process.

Studies comparing (a) non-demented individuals free of substantial Alzheimer’s pathology (controls), (b) non-demented individuals with equivalent loads of amyloid-β plaques (“mismatches”) and tangles, and (c) demented Alzheimer’s cases, observed four main phenotypic differences between the groups, which include demented cases had significantly higher burdens of fibrillar thioflavin-S positive plaques and of oligomeric amyloid-β deposits reactive to conformer-specific antibody NAB61 than “mismatches”.[32] Thus, florbetapir PET most likely could not distinguish between these different forms of amyloid deposition. Therefore, future amyloid phenotype differentiating the pathological forms of amyloid deposition will help genetic association study.

Future studies with larger sample sizes or replication samples are needed to further dissect the genetic basis of CSF and cerebral biomarkers. AD progression is a challenging problem as the patients are likely to be at different stages of the disease continuum, additionally, deterioration is not linear over the course of the disease progression. Furthermore, different neuropsycogntive instruments are sensitive for patients at different stages of the disease continuum, thus, genetic association studies further suffers from the sample size after stratification by disease stage.

## Methods

### Alzheimer’s Disease Neuroimaging Initiative

Data used in this study were obtained from the ADNI database (adni.loni.usc.edu). The ADNI study was launched in 2003 by the National Institute on Aging (NIA), the National Institute of Biomedical Imaging and Bioengineering (NIBIB), the Food and Drug Administration (FDA), private pharmaceutical companies and non-profit organizations, as a $60 million dollar, 5-year public private partnership. The primary goal of ADNI study has been to test whether serial magnetic resonance imaging (MRI), positron emission tomography (PET), other biological markers, and clinical and neuropsychological assessments can be combined to measure the progression of MCI and early AD. For up-to-date information, see www.adni-info.org.

The data from ADNIMERGE R package dated 2014-06-11 together with genetic data (Figure A in S1 File) (http://www.loni.ucla.edu/ADNI) were utilized in this manuscript. The adnimerge table merges together several of the key variables from various case report forms and biomarker lab summaries across the ADNI protocols (ADNI1, ADNIGO, and ADNI2). The details of genotype data quality control (QC), imputation, and genetic association analysis are described in the S1 File.

### Inference of APOE ε4 genotype

A fraction of subjects with OmniExpress genotype data had missing APOE ε4 dosage in the ADNIMERGE database. Genotype dosage for rs429358 which is the defining variant for APOE ε4 dosage were imputed using IMPUTE2 [33–37] (v2.3.0) and the rs429358 genotype was inferred using the best guessed genotype if the probability having that best guessed genotype exceeds 90%.

### CSF biomarker GWAS meta-analysis

To minimize differences due to different CSF assay batches, three sets of samples (Table 1) were used in the GWAS and referred to as Upennbiomk_Human610-Quad, Upennbiomk5_Omni2.5, and Upennbiomk6_OmniExpress. Sample sizes for CSF biomarkers after sample level QC and with phenotype data are listed in Table A in S1 File. CSF biomarker data at baseline visit of ADNI study were log transformed to approximate a normal distribution. *APOE* ε4 allele dosage, age and clinical diagnosis group (CN, EMCI, LMCI, or AD) at baseline visit were included as covariates. Fixed effects meta-analyses were carried out using PLINK.[38] Regional visualization of genome-wide association scan results was plotted using LocusZoom.[39]

### Cerebral amyloid deposition (florbetapir PET) quantitative trait GWAS

The cerebral amyloid deposition quantitative trait as measured by florbetapir PET was taken from adnimerge table. The AV45 value in the adnimerge table is the average AV45 SUVR of frontal, anterior cingulate, precuneus, and parietal cortex relative to the cerebellum. Independent GWA analyses using florbetapir PET SUVR as a quantitative trait were performed for subjects genotyped with the Omni2.5 (N = 661) or the OmniExpress (N = 291) platform (see Table B in S1 File for basic demographic characteristics), followed by meta-analysis across the two sample sets. For patients with more than one florbetapir PET imaging data, the peak measurement was used in this analysis. Gender, age and clinical diagnosis (NL, MCI, or AD) at the time of peak florbetapir PET measurement were included as covariates. Other auxiliary analyses are described in the S1 File.

### Rate of cognitive decline GWAS

LMCI subjects (N = 540) with genetic data were included in this analysis. The subset of overlapping markers shared across all samples was used to impute unobserved genotype data since chip platform is correlated with rate of cognitive decline to avoid the situation of using different sets of markers to infer unobserved genotyping being confounded with the phenotypic endpoint. The rate of cognitive decline is defined by the yearly rate of CDR-SB score change over one of the following: (1) the first two year period since baseline (if month 24 data are available); or (2) the first one year (if month 24 data are not available but month 12 data are available); or (3) the first 6 month (if only month 6 data are available). The rate of cognitive decline was also log transformed after addition of 3.5 to avoid log transformation of negative numbers using this formula ln (ΔCDRSB/duration + 3.5) to approximate normal distribution. Gender, age, baseline CDR-SB score, baseline MMSE score, and *APOE* ε4 allele dosage were included as covariates.

### Gene Set Enrichment Analysis

INRICH is a pathway analysis tool for genome wide association studies, designed for detecting enriched association signals of LD-independent genomic regions within biologically relevant gene sets.[15] Reference gene sets used in the INRICH analysis include KEGG, Gene Ontology, and Molecular Signature Database. Top variants from CSF and florbetapir PET SUVR analyses with nominal association p-values less than 0.0005, 0.0001, 0.00005, 0.00001 were separately fed into PLINK to clump the variants into LD-independent genomic intervals (r^2^ threshold using 0.2, 0.3, and 0.5 respectively), then LD-independent genomic regions were used for INRICH (version 1.0) analyses. No multiple testing correction was applied for running INRICH against multiple reference gene sets or for using multiple parameters (p-value cut-off and LD threshold).

## Supporting Information

S1 File Supporting Information. (DOCX)

S1 Table Full list of variants with uncorrected p-value less than 1x10^-6^ in any CSF biomarker analyses. (XLSX)

S2 Table Full list of variants with uncorrected p-value less than 1x10^-6^ in florbetapir PET quantitative trait and dichotomized trait analyses. (XLSX)

S3 Table Full list of variants with uncorrected p-value less than 1x10^-6^ in Cerebral amyloid deposition florbetapir PET quantitative traits stratified by ε4 carrier status and amyloid positivity dichotomized traits. (XLSX)

S4 Table Full list of variants with uncorrected p-value less than 1x10^-6^ in rate of cognitive decline analyses among LMCI patients. (XLSX)

S5 Table Full list of Inrich analysis results. (XLSX)
