# Supplementary material for: Variations in the FRA10AC1 Fragile Site and 15q21 Are Associated with Cerebrospinal Fluid Aβ1-42 Level
Source: PLoS One. 2015 Aug 7;10(8):e0134000. doi: 10.1371/journal.pone.0134000 (PMC4529186; doi:10.1371/journal.pone.0134000)
Supplement: S1 File — (DOCX) [file pone.0134000.s001.docx]

Variations in the *FRA10AC1* Fragile Site and 15q21 are Associated with Cerebrospinal Fluid Aβ_1-42_ Level

Qingqin S. Li^1^, Antonio R. Parrado^2^, Mahesh N. Samtani^3^, and Vaibhav A. Narayan^1^ for the Alzheimer’s Disease Neuroimaging Initiative*

^1^Neuroscience Therapeutic Area, Janssen Research & Development, LLC, 1125 Trenton-Harbourton Road, Titusville, NJ 08560, USA

^2^Discovery Science, Janssen Research & Development, LLC, 1400 McKean Road, Spring House, PA 19002, USA

^3^Clinical Pharmacology, Advanced PK/PD Modeling and Simulation, Janssen Research & Development, LLC, 920 Route 202, Raritan, NJ 08869, USA

Address correspondence and reprint requests to Dr. Qingqin S. Li, Neuroscience Therapeutic Area, Janssen Research & Development, LLC, 1125 Trenton-Harbourton Road, Titusville, NJ 08560, USA [qli2@its.jnj.com](mailto:qli2@its.jnj.com)

*Data used in preparation of this article were obtained from the Alzheimer’s Disease Neuroimaging Initiative (ADNI) database (adni.loni.usc.edu). As such, the investigators within the ADNI contributed to the design and implementation of ADNI and/or provided data but did not participate in analysis or writing of this report. A complete listing of ADNI investigators can be found at:

http://adni.loni.usc.edu/wp-content/uploads/how_to_apply/ADNI_Acknowledgement_List.pdf

**Supporting Information**

**ADNI Study Data**

### Alzheimer’s Disease Neuroimaging Initiative. Data used in this study were obtained from the ADNI database (adni.loni.usc.edu). The ADNI study was launched in 2003 by the National Institute on Aging (NIA), the National Institute of Biomedical Imaging and Bioengineering (NIBIB), the Food and Drug Administration (FDA), private pharmaceutical companies and non-profit organizations, as a $60 million dollar, 5-year public private partnership. The primary goal of ADNI study has been to test whether serial magnetic resonance imaging (MRI), positron emission tomography (PET), other biological markers, and clinical and neuropsychological assessments can be combined to measure the progression of MCI and early AD. Determination of sensitive and specific biomarkers of very early AD progression is intended to aid researchers and clinicians to develop new treatments and monitor their effectiveness, as well as lessen the time and cost of clinical trials.

### The Principal Investigator of this initiative is Michael W. Weiner, MD, VA Medical Center and University of California, San Francisco. The ADNI study is the result of efforts of many co-investigators, from a broad range of academic institutions and private corporations, who have recruited subjects from over 50 sites across the U.S. and Canada. The initial goal of the ADNI study was to recruit 800 subjects, however ADNI co-investigators have been ambitious by extending the study to ADNI-GO and ADNI-2. To date these three research protocols have recruited over 1500 adults, ages 55 to 90, consisting of cognitively normal older individuals, people with early or late MCI, and people with early AD. The follow up duration of each group is specified in the protocols for ADNI-1, ADNI-2 and ADNI-GO. Subjects originally recruited for ADNI-1 and ADNI-GO had the option to be followed in ADNI-2. For up-to-date information, see [www.adni-info.org](http://www.adni-info.org).

Specifically, the subsets of ADNI data utilized in our analyses:

1) adnimerge data frame obtained using ADNIMERGE_0.0.1.tar.gz (dated 2014-06-12 though adnimerge.rdata dated 2014-06-11; the dates of *.rdata were referenced in the manuscript).

2) ADNI CSF biomarker data presumably identical to upennbiomk/2/3/4/5/6/7 R data frames in ADNIMERGE database: seven batches of CSF biomarker data were available from ADNI database (adni.loni.usc.edu) for download at the time of this study and they are UPENN-Biomarker Data upennbiomk.csv (identical to upennbiomk.rdata) consisting of baseline Aβ_1-42_, p-tau_181p_, and t-tau for 415 ADNI1 subjects (+1 screening failed subject: RID=975), UPENN-Longitudinal Data (UPENNBIOMK2.csv), UPENN-Longitudinal Data (3yr) (UPENNBIOMK3.csv), UPENN-Longitudinal Data (4yr) (UPENNBIOMK4_09_06_12.csv), UPENN-CSF Biomarkers [ADNI-GO/-2] (UPENNBIOMK5_10_31_13.csv, identical to upennbiomk5.rdata) consisting of baseline Aβ_1-42_, p-tau_181p_, and t-tau for 117 ADNI-GO subjects and 272 ADNI-2 subjects, UPENNBIOMK6_07_02_13.csv, and UPENNBIOMK7 (2014-06-09) - Third Batch Analysis of CSF Biomarkers [ADNI GO/2], respectively;

3) ADNI genetic data: four batches of genetic data were available from ADNI database (adni.loni.usc.edu) for download at the time of this study and they are ADNI-1 samples genotyped using the Illumina Human610-Quad BeadChip, ADNI-GO/-2 samples genotyped using the Illumina HumanOmniExpress BeadChip, ADNI-1/-GO/-2 Whole Genome Sequencing (WGS) samples genotyped using the Illumina Omni2.5 BeadChip, and a second wave of ADNI-GO/-2 samples genotyped using the Illumina HumanOmniExpress BeadChip released on 2014-08-05 (Figure A). For the disease risk endpoint and some of the auxiliary cerebral amyloid deposition sub-analyses reported in the manuscript, only the first three batches of GWAS genetic data were included.

### Genotype data quality control (QC), imputation, and genetic association analysis:

### For each of the GWAS analysis described below, genotype data from different platforms were filtered on both subject and SNP level, separately. We only included SNPs with genotype missing rates < 5%, minor allele frequency (MAF) > 1%, and Hardy–Weinberg equilibrium (HWE) *P* value > 0.000001. We excluded from further analysis any sample that had missing genotypes for more than 2% of the SNPs on the array. Additionally, we generated pairwise identity-by-descent (IBD) values for all samples using the PLINK (v1.07)[^1^](#_ENREF_1), excluding one sample from any pair of duplicate samples or samples with cryptic relatedness (i.e. with a PI_HAT value exceeding 0.3). Principal components were generated on each cohort using EIGENSTRAT[^2^](#_ENREF_2)^,^[^3^](#_ENREF_3) using default parameters except adding the options of nsnpldregress: 3 and maxdistldregress: 1. Population outliers were removed from subsequent genetic association analysis unless specified otherwise.

To directly compare variants via meta-analysis across the cohorts genotyped using different Illumina platforms, thus allowing the exploration of a greater density of markers than what was genotyped directly, we imputed genotypes using IMPUTE2 (v2.3.0).[^4-8^](#_ENREF_4) Imputation of unobserved genotypes was based on the reference haplotypes from the 1,000 Genomes haplotypes; Phase I integrated variant set release (SHAPEIT2) in NCBI build 37 (hg19) coordinates[^9^](#_ENREF_9), singletons from the 1000 Genomes Project (1,092 individuals) were removed from autosomes. Internal cross validation was carried out automatically by IMPUTE2. The calculation was performed by masking one variant at a time in the study data, imputing the masked variant and comparing the result to the original genotype. Concordance for variants with maximum posterior probability > 90% (interval [0.9-1.0]) was > 96%. Both the directly genotyped markers and imputed dosage genotypes were assessed for association in a linear or logistic regression model, correcting for significant covariates and/or covariates included in reported GWAS together with principal components to account for population substructure. For SNP**APOE* ε4 interaction analysis, only the directly genotyped markers were utilized. Genomic Inflation Factors (GIF) in directly genotyped datasets before and after population outlier removal and ancestry principal component correction were listed in Table A., We filtered imputed variants with r-squared quality metric / information content > 0.3 for association analysis, a total of ~9M imputed variants were examined. A conventional p-value threshold of 5x10^-8^ was used to declare genome-wide significance. A suggestive significance threshold at 1x10^-6^ was used to report the summary results. No correction was made to account for multiple testing across the multiple analyzed cohorts. Manhattan and QQ-plots were generated using the R package gap[^10^](#_ENREF_10) and regional plots were created using LocusZoom (v1.2).[^11^](#_ENREF_11)

**Baseline CSF biomarker GWAS meta-analysis:**

This study contains downloaded data from the ADNI database which included 1,131 subjects with CSF biomarker data from three batches (upennbiomk1/5/6).

1. 391 unique subjects with (upennbiomk) CSF data and genotyped using Human610-Quad BeadChip,
2. 385 unique subjects with (upennbiomk5) CSF data and genotyped using Omni2.5 BeadChip, and
3. 204 unique subjects with (upennbiomk6) CSF data and genotyped using OmniExpress BeadChip (5 unique samples from Sample B & Sample I in Figure A) (Table 1).

In addition, a SNP**APOE* ε4 interaction analysis was performed for baseline CSF Aβ_1-42_ level for directly genotyped markers only.

**Cerebral amyloid deposition (florbetapir PET) quantitative trait GWAS:**

This download of the ADNI database included 968 subjects of European ancestry with florbetapir PET imaging data, of which 661 were genotyped with the Illumina Omni2.5, 291 with the OmniExpress, and 170 with the Illumina 610-Quad (164 of the 170 subjects were also genotyped with the Omni2.5), and 10 subjects were not genotyped yet. The florbetapir PET imaging measurement was taken at baseline for new ANDI-2 participants, at baseline and/or month 24 for ADNI-GO participants, and at month 60, 72, and/or 84 for ADNI-1 participants continuing into ADNI-GO/-2. Sample sizes for CSF biomarkers and florbetapir PET after sample level QC are listed in Table A.

In addition to the primary meta-analysis which did not correct for *APOE* ε4 allele dosage, we carried out a sub-analysis including *APOE* ε4 allele dosage as a covariate, in the Omni2.5 cohort only, to identify loci independent of *APOE* ε4. This sub-analysis was not carried out in the OmniExpress genotyped cohort because of the significant amount of missing *APOE* ε4 data. Furthermore to identify variants that might have significant SNP**APOE* ε4 interaction in the Omni2.5 cohort, an exploratory analysis was carried out for *APOE* ε4-carriers and ε4-noncarriers separately, and a formal SNP**APOE* ε4 interaction analysis on directly genotyped markers was performed for florbetapir PET.

**Cerebral amyloid deposition (florbetapir and PIB PET) dichotomized Aβ positivity GWAS:**

Patients were defined as Aβ positive using PET imaging alone (florbetapir SUVR > 1.1 or PIB SUVR > 1.5 at any time point). The GWAS analyses were performed with all subjects irrespective of their *APOE* ε4 genotype status and separately among *APOE* ε4 non-carriers. No clinical covariate (i.e. gender, age, or clinical diagnosis) was adjusted for in these analyses.

**Rate of cognitive decline** **GWAS**:

The 2014-06-11 download of the ADNI database included 416 CN, 106 SMC, 311 EMCI, 563 LMCI, and 340 AD patients. The LMCI patient population, because of largest sample size, was used to study the rate of cognitive decline. Among the 563 LMCI subjects, 23 of them do not have GWAS genetic data. To avoid confounding by the phenotypic endpoint, since chip platform is correlated with rate of cognitive decline, an overlapping subset of markers shared across all samples was used to impute unobserved genotype data, thus, avoiding the situation of using different sets of markers for imputation. Furthermore, there were only 68 unique subjects genotyped using HumanOmniExpress, 248 subjects genotyped using Omni 2.5 M, and 364 subjects genotyped using Human610-Quad (140 of which also had Omni 2.5 M data). Each cohort is small and therefore we did not adopt the approach of analyzing each cohort by genotyping platform separately followed by meta-analysis. Instead, the overlapping set of variants was used to impute genotypes. Table A contains sample sizes for disease progression after sample level QC.

**Top CSF Variant rs10509663 result from florbetapir PET analysis and other endpoints**

Using florbetapir PET as a quantitative trait endpoint, we observed a nominal association for the top variant associated with baseline CSF Aβ_1-42_ rs10509663 and SUVR value (*P* = 0.01) among the *APOE4* ε4 non-carriers but not in the full samples with or without correction for *APOE* ε4 dosage or among the *APOE4* ε4 carriers (*P* > 0.05). The interaction analysis with rs10509663-G dosage**APOE* ε4 dosage confirmed that there was a nominal statistical interaction (*P*_interaction_ = 0.03 with main effects *P*_ε4_ = 3.82 x 10^-24^ and *P*_rs10509663_ = 0.02). When dichotomizing florbetapir and PIB SUVR values to define Aβ positivity, we observed a nominal association (*P* = 0.05) between the rs10509663 and Aβ positivity, interestingly the association is a bit stronger (*P* = 0.006) in the Aβ positivity analysis among the *APOE4* ε4 non-carriers, consistent with the results from the florbetapir PET quantitative trait analysis. Given the nominal statistically significant interaction between rs10509663-G dosage and *APOE* ε4 dosage, we also tested the statistical interaction in CSF Aβ data and it was marginally significant with *P*_interaction_ = 0.06, main effects P_ε4_ = 2.0 x 10^-26^ and P_rs10509663_ = 1.68 x 10^-6^ in the Upennbiomk5_Omni2.5 cohort, and P_interaction_ = 0.009, main effects P_ε4_ = 6.18 x 10^-21^ and P_rs10509663_ = 1.63 x 10^-5^ in the Upennbiomk_Human610-Quad. We did not observe a nominal association between rs10509663 and clinical diagnosis among *APOE4* ε4 non-carriers (*P* = 0.42). We did not observe a significant association (*P* = 0.27 European ancestry cohort, *P* = 0.13 cohort of all races for rs10509663; *P* = 0.88 European ancestry cohort and *P* = 0.48 cohort of all races for rs4301994) between the two genome-wide significant CSF variants and rate of cognitive decline in the LMCI cohort. Graphical exploration of rs10509663 in AD subgroups also showed no trend of separation by genotype status.

**GWAS results from the florbetapir PET analyses**

There are a few relatively common (rs12154229, an intergenic variant between small lysine-rich protein 1 (*SMKR1*), and nuclear respiratory factor 1 (*NRF1*), *P* = 6.18 x 10^-7^ correcting for *APOE* ε4 dosage; rs28810, an intronic variant in N(alpha)-acetyltransferase 60, NatF catalytic subunit (*NAA60*), *P* = 4.78 x 10^-7^ without correction for *APOE* ε4 dosage, *P* = 2.76 x 10^-5^ with correction for *APOE* ε4 dosage) and uncommon variants (rs76117213, an intronic variant in WD repeat and FYVE domain containing 3 (*WDFY3*), *P* = 1.39 x 10^-7^ without correction for *APOE* ε4 dosage, *P* = 1.93 x 10^-7^ with correction for *APOE* ε4 dosage; rs139095266, an intronic variant in thioredoxin-related transmembrane protein 3 (*TMX3*), *P* = 7.76 x 10^-8^ among *APOE* ε4 non-carriers; and rs140320399, an intronic variant in guanine nucleotide binding protein (G protein), gamma 4 (*GNG4*), *P* = 5.23 x 10^-8^ among *APOE* ε4 non-carriers) with suggestive association in other florbetapir PET GWAS sub-analyses. The uncommon variant results, however, shall be interpreted with caution as these variants occurred at low minor allele frequency and the association statistics are based on small genotype groups.

**Results from this study for variants reported in the literature:**

The CSF GWAS study by Cruchaga et al.,[^13^](#_ENREF_13) identified rs9877502 (*P* = 4.89 x 10^-9^ for t-tau) located at 3q28 between *GEMC1* and *OSTN*, rs514716 (*P* = 1.07 x 10^-8^ and *P* = 3.22 x 10^-9^ for t-tau and p-tau_181p_) located at 9p24.2 within *GLIS3* and rs6922617 (*P* = 3.58 x 10^-8^ for CSF p-tau_181p_) at 6p21.1 within the *TREM* gene cluster, a region reported to harbor rare AD risk variants. Variant rs9877502 also showed suggestive association with risk for AD, tangle pathology, and global cognitive decline (p = 2.67 x 10^-4^, 0.039, 4.86 x 10^-5^, respectively) in independent datasets suggesting that endophenotype-/quantitative trait-based approach could be used to identify novel AD risk loci. All three variants showed suggestive association in our Upennbiomk_Human610-Quad cohort, which was also included in the reported CSF studies (rs9877502 *P* = 1.57 x 10^-5^ for t-tau and *P* = 0.002 for p-tau_181p_, rs514716 *P* = 0.06 for t-tau and *P* = 0.07 for p-tau_181p_, and rs6922617 *P* =0.0003 for t-tau and *P* = 9.73 x 10^-5^ for p-tau_181p_) but the Upennbiomk5_Omni2.5 cohort did not provide additional supporting evidence for rs9877502 (*P* = 0.36 for t-tau and p-tau_181p_), rs514716 (*P* = 0.76 for t-tau and *P* = 0.32 for p-tau_181p_), and rs6922617 (*P* = 0.87 for t-tau and *P* = 0.36 for p-tau_181p_). Upennbiomk6_OmniExpress cohort provided partial supporting evidence for rs9877502 (*P* = 0.22 for t-tau and *P* = 0.01 p-tau_181p_, the direction being consistent with the Upennbiomk_Human610-Quad cohort), and no supporting evidence for rs514716 (*P* = 0.43 for t-tau and *P* = 0.59 for p-tau_181p_) and rs6922617 (*P* = 0.70 for t-tau and *P* = 0.81 for p-tau_181p_). The latest large scale genetic study identified 19 novel disease risk variant via a two-stage design study, 17,008 AD cases and 37,154 controls were meta-analyzed in stage 1, and 11,632 SNPs were genotyped and tested for association in an independent set of 8,572 AD cases and 11,312 controls in stage 2.[^12^](#_ENREF_12) We sought to provide replication of these variants in our analysis, interestingly, a subset of them showed nominal association with CSF biomarker. The most significant variant was rs10498633 from the *SLC24A4-RIN3* gene that showed suggestive association with Aβ_1-42_ level (p = 0.01).

Rs509208 near *BCHE* (butyrylcholinesterase) previously identified as a hit, passing the conventional genome-wide significant threshold, showed suggestive association with florbetapir^[14](#_ENREF_14)^ (*P*_FE_ = 0.0003, Omni2.5 cohort *P* = 1.84 x 10^-5^ and OmniExpress cohort *P* = 0.85) in our analysis. The additional genetic data from Omni2.5 and OmniExpress did not strengthen and in fact weaken the florbetapir PET association reported for rs509208.[^14^](#_ENREF_14) Several reasons attributed to our results: 1) the original analysis of 555 subjects with PET data was based on data from Illumina 610-Quad or OmniExpress (Sample A and B in the Figure A). Minimac instead of IMPUTE2 was used to impute samples within groups based on the genotyping platform employed and the independently-imputed data sets were then merged for genetic association. In our analysis, samples genotyped on different genotyping platform were imputed and used in the GWAS independently followed by meta-analysis; 2) the OmniExpress 2.5M samplesshould overlap substantially with the reported 555 subject cohort as all but five samples genotyped on OmniExpress (Sample B in Figure A) and most of the subjects with PET imaging data and genotyped on 610-Quad were also genotyped on Omni2.5. The difference in imputed genotypes based on different marker space and the minor difference in sample size results in the difference of the reported 2.69 × 10^-8^ association p-value and the observed 1.84 x 10^-5^ from the Omni2.5 cohort analysis; 3) the additional samples genotyped on OmniExpress cohort (Sample I and the unique subjects from Sample B in Figure A) unfortunately did not support the association (*P* = 0.85) in this analysis.

The study by Hu et al., (2011), consisting of 489 MCI subjects from a Vitamin E trial and 333 ADNI MCI subjects, identified three variants (rs7840202 on chromosome 8 in *UBR5*: *P* = 4.27 x 10^-14^; rs11637611 within a cluster of SNPs on 15q23 close to the Tay–Sachs disease locus: *P* = 1.07 x 10^-15^; and rs12752888 on chromosome 1: *P* = 3.08 x 10^-11^) associated with a significant decline in cognition based on repeat measures analysis of variance (significant time x genotype interaction).[^15^](#_ENREF_15) Although the statistical model used by Hu et al., is different from the one we used in our analysis, and noting there is a partial sample overlap, our analysis yielded a nominal association (rs7840202 *P* = 0.02, rs11637611 *P* = 0.056 in European ancestry LMCI cohort, and P= 0.02 and P = 0.056 the LMCI cohort of all races) and non-significant for rs12752888 (*P* = 0.68 in the European ancestry LMCI cohort and *P* = 0.85 for the LMCI cohort of all races).Our analysis did not take non-linear response into consideration and assumed the early progression (up to month 24) was linear and it might contribute to the difference.

**Figure A: Subject and SNP Overlaps between the four batches of ADNI genetic data**.

1. Overlaps between ADNI samples (A) ADNI-1 samples genotyped using the Illumina Human610-Quad BeadChip, (B) ADNI-GO/-2 samples genotyped using the Illumina HumanOmniExpress BeadChip, (C) ADNI-1/-GO/-2 Whole Genome Sequencing (WGS) samples genotyped using the Illumina Omni2.5 BeadChip, (I) another wave of ADNI-GO/-2 samples genotyped using the Illumina HumanOmniExpress BeadChip; 5 unique subjects from Sample B were pooled with subjects from Sample I.
2. Overlaps between SNPs (D) Illumina Human610-Quad BeadChip with 655,214 variants on the chip, (E) Illumina HumanOmniExpress BeadChip with 731,442 variants on the chip, (F) Illumina Omni2.5 BeadChip with 2,391,739 variants on the chip.

1)


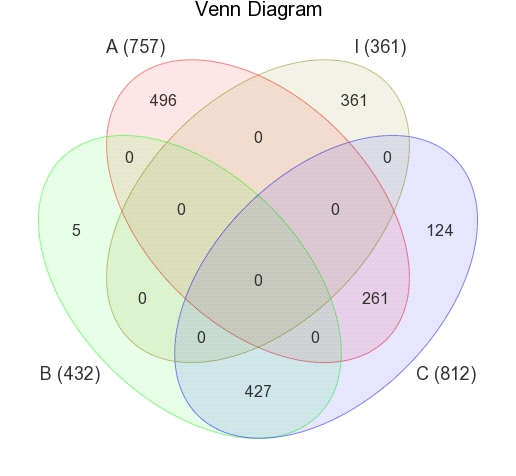


2)


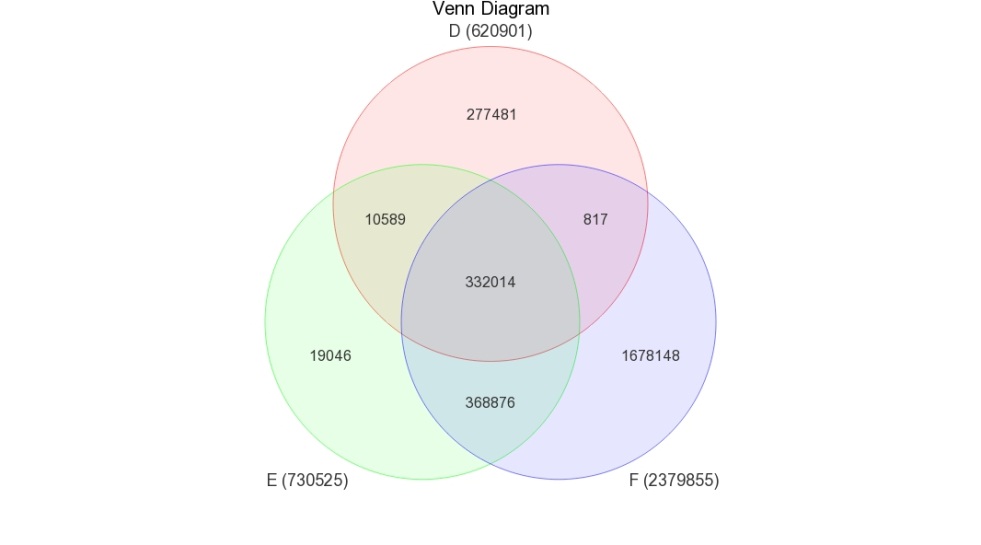


**Figure B: Manhattan plots of the CSF Biomarker GWAS Meta-Analyses.**

(1) Aβ_1-42_ discovery phase, (2) t-Tau joint analysis, (3) p-Tau_181P_ joint analysis, (4) t-Tau:Aβ_1-42_ ratio joint analysis, (5) p-Tau_181P_:Aβ_1-42_ ratio joint analysis**.** The dotted line indicates genome wide significance threshold of 5x10^-8^. Only variants with p < 0.05 are shown.

(1)


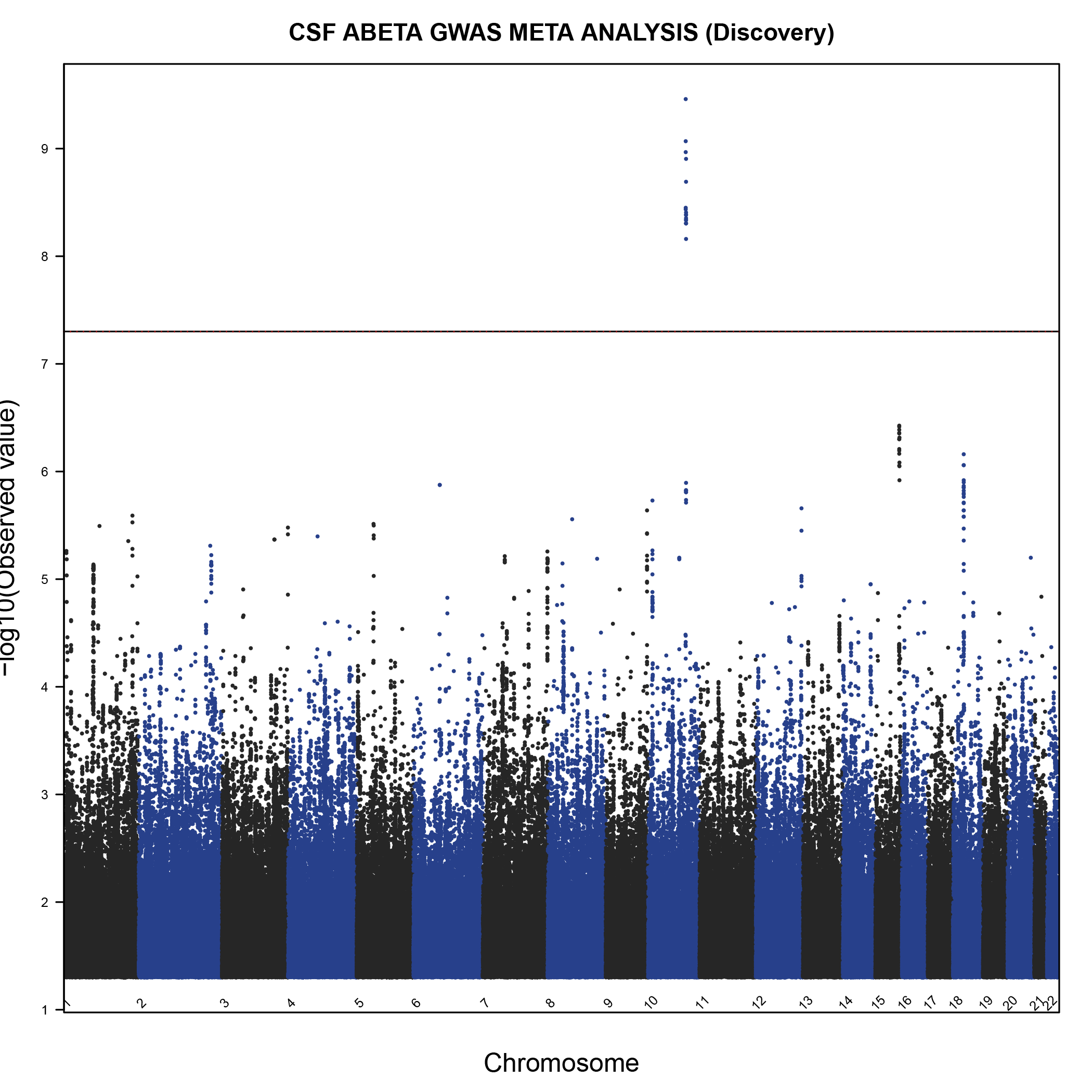


(2)


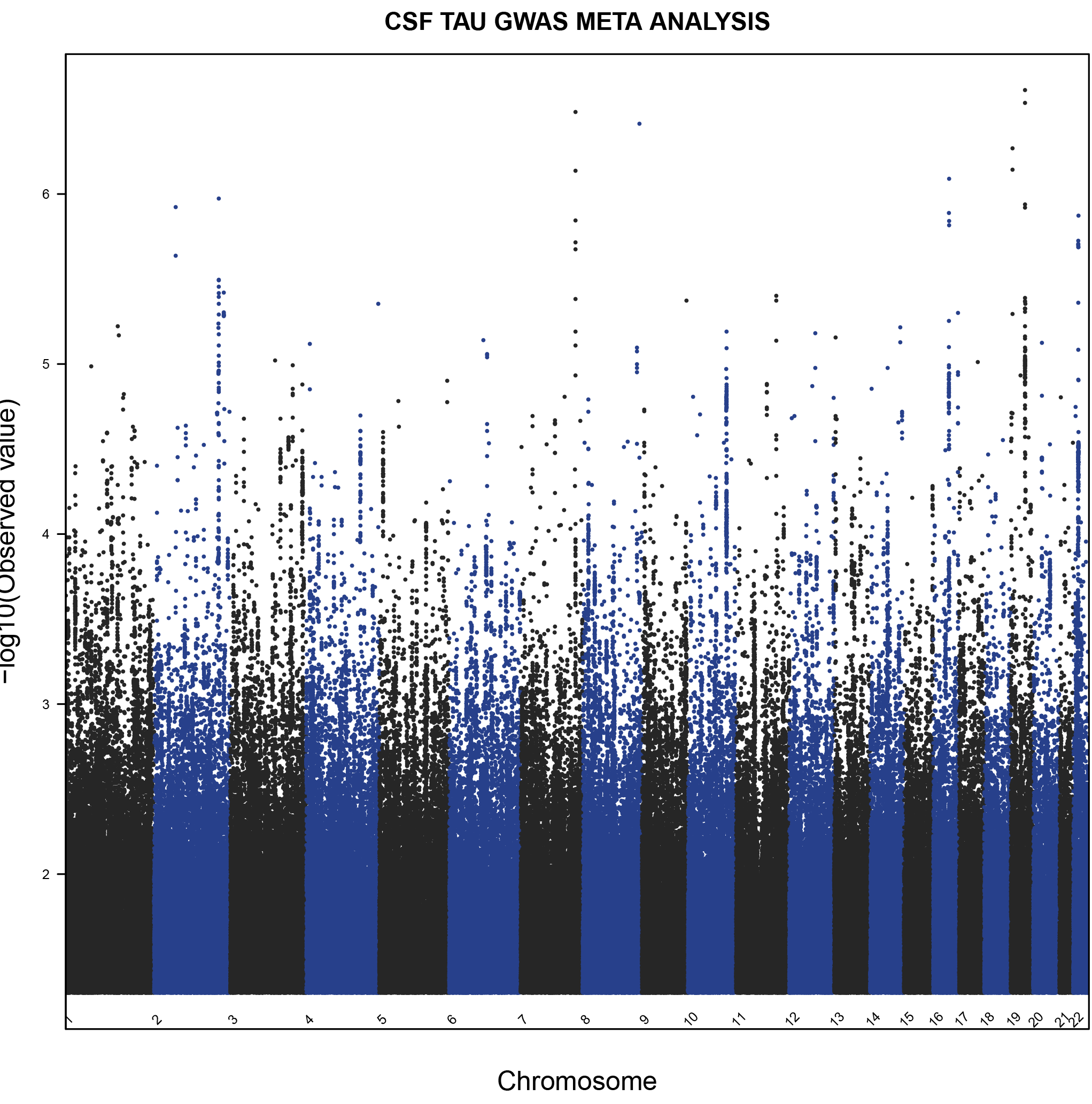


(3)


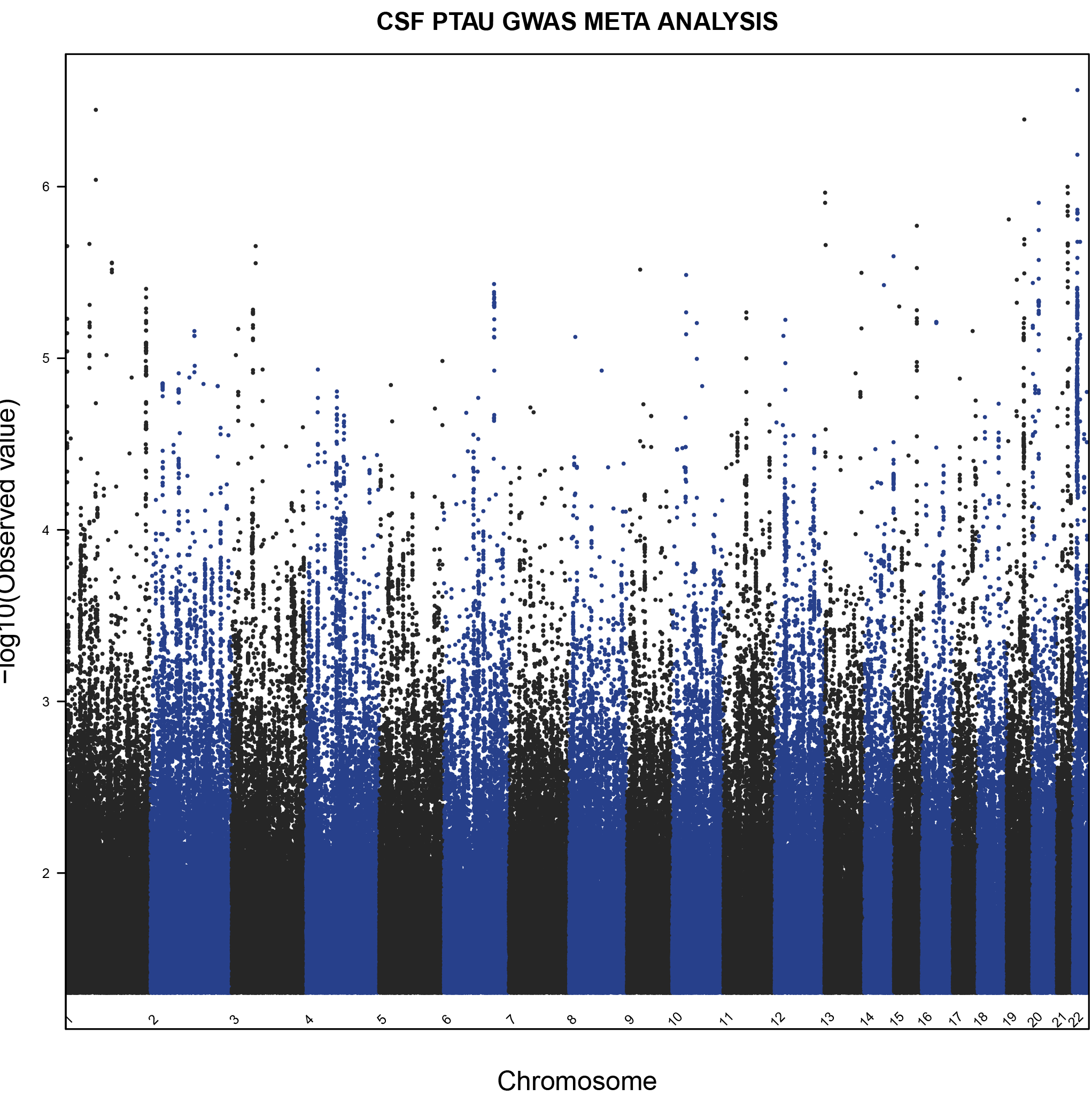


(4)


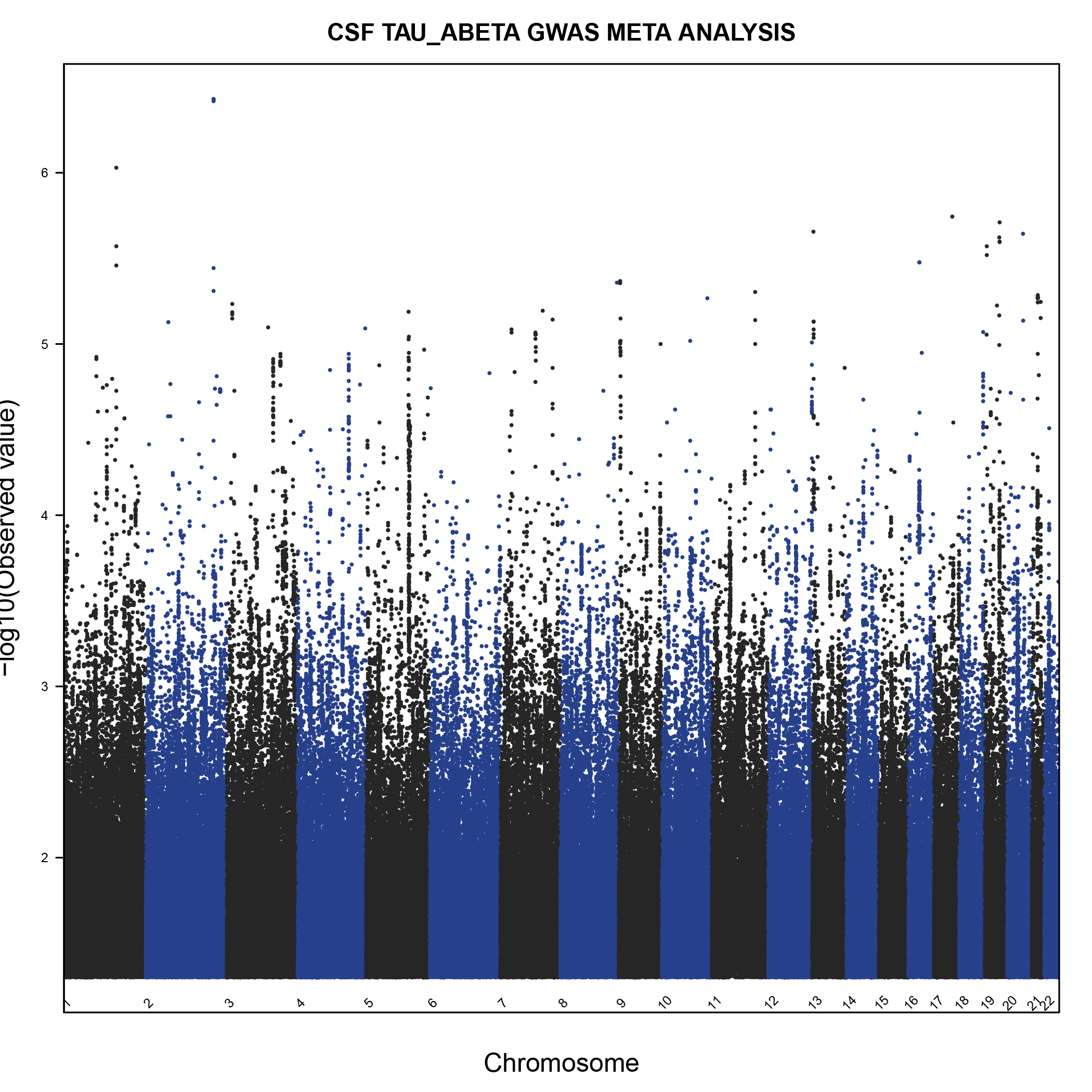


(5)


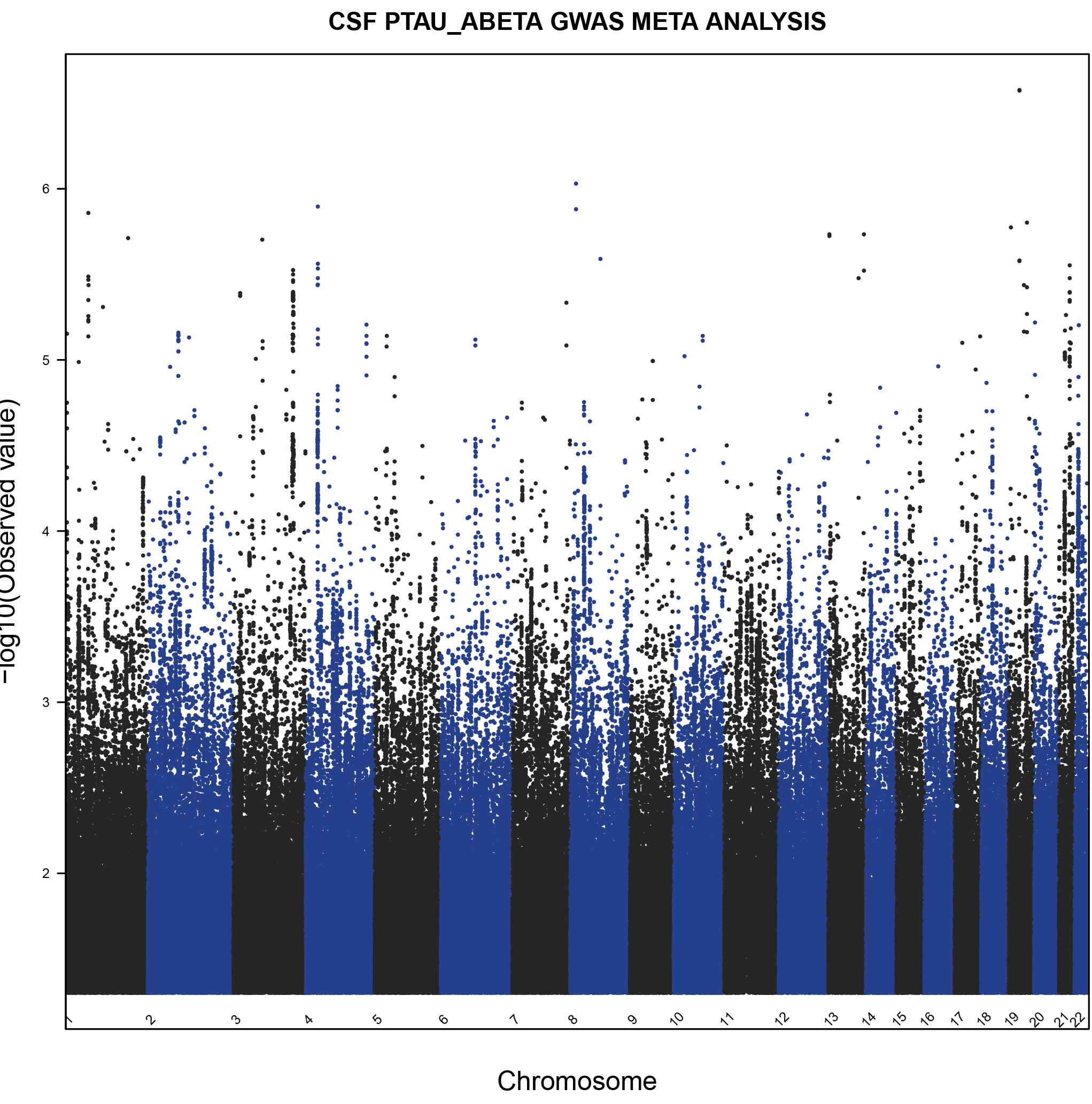


**Figure C: Manhattan plots of florbetapir PET quantitative traits (1)-(4) and dichotomized traits (5)-(6).** (1) AV45 SUVR meta-analysis without correcting for *APOE* ε4 dosage, (2) AV45 SUVR GWAS correcting for *APOE* ε4 dosage, (3) AV45 SUVR GWAS among *APOE* ε4 carriers, (4) AV45 SUVR GWAS among *APOE* ε4 non-carriers, (5) Amyloid PET positivity GWAS, and (6) Amyloid PET positivity GWAS among *APOE* ε4 non-carriers**.** The dotted line indicates genome wide significance threshold of 5x10^-8^. Only variants with p < 0.05 are shown.

**(1)**


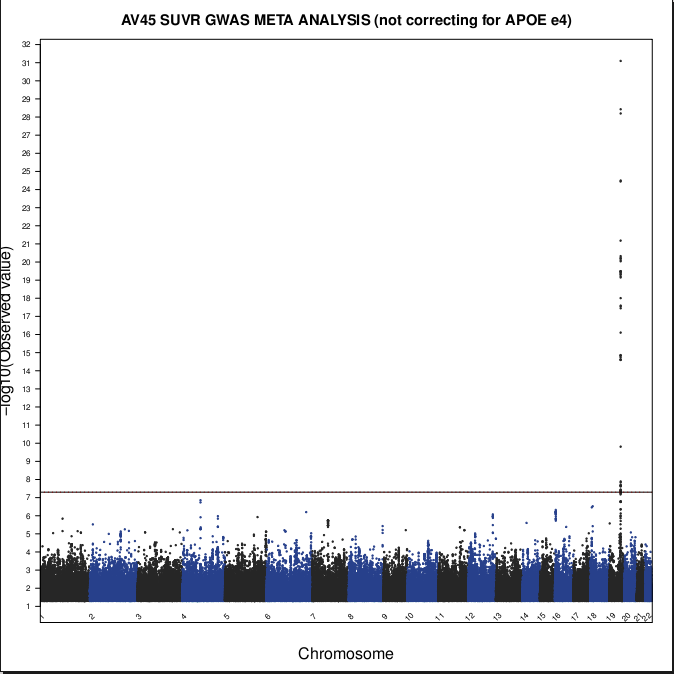


(2)

**
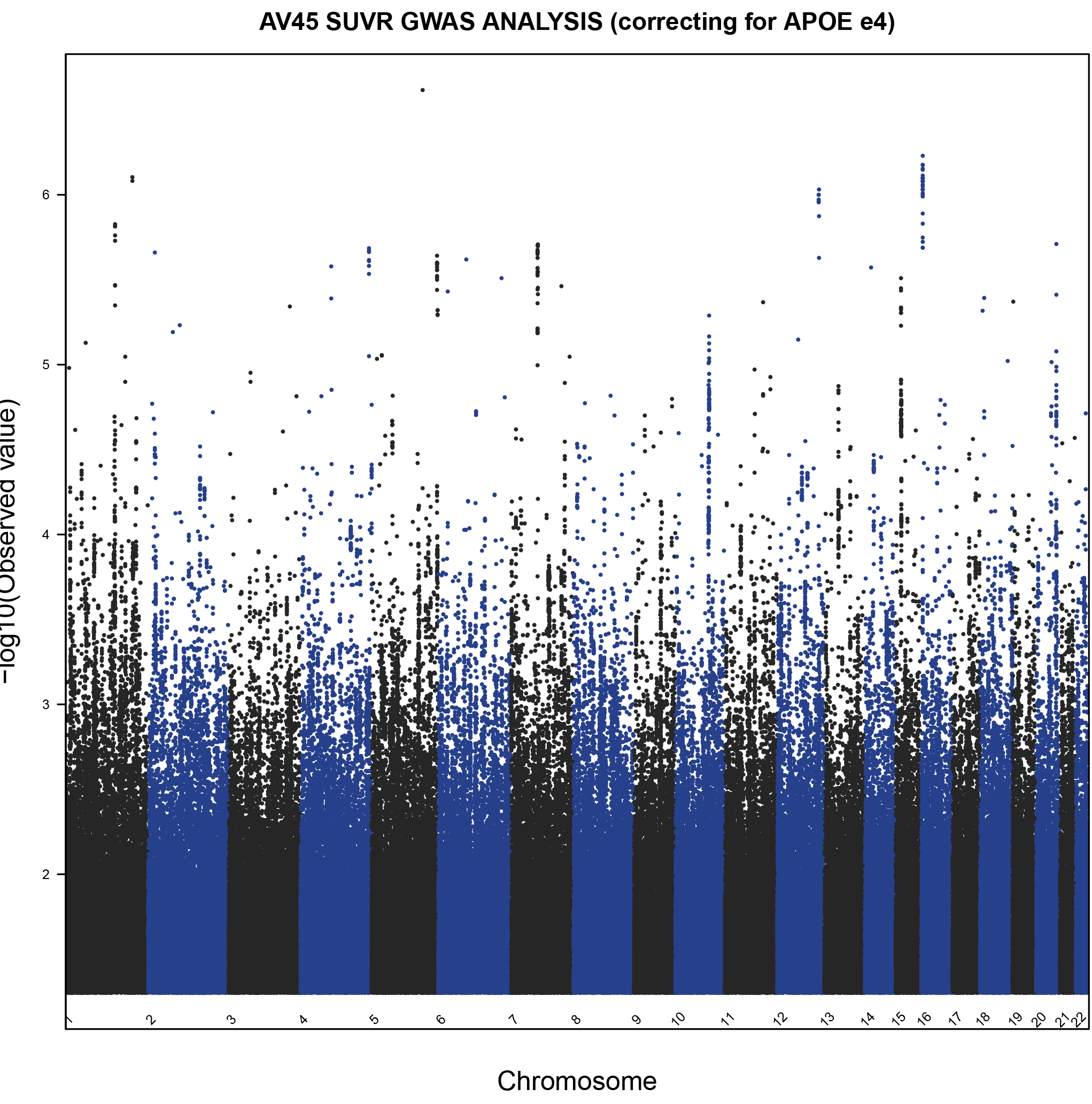
**

(3)


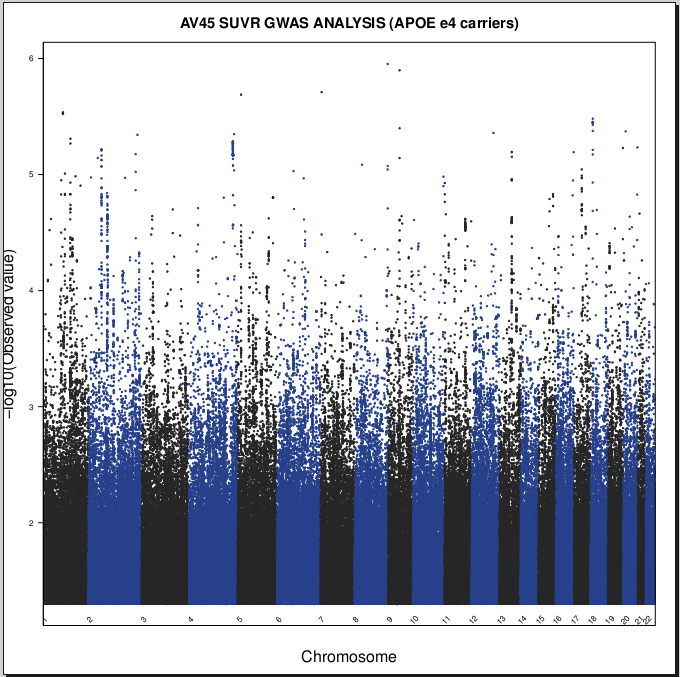


(4)


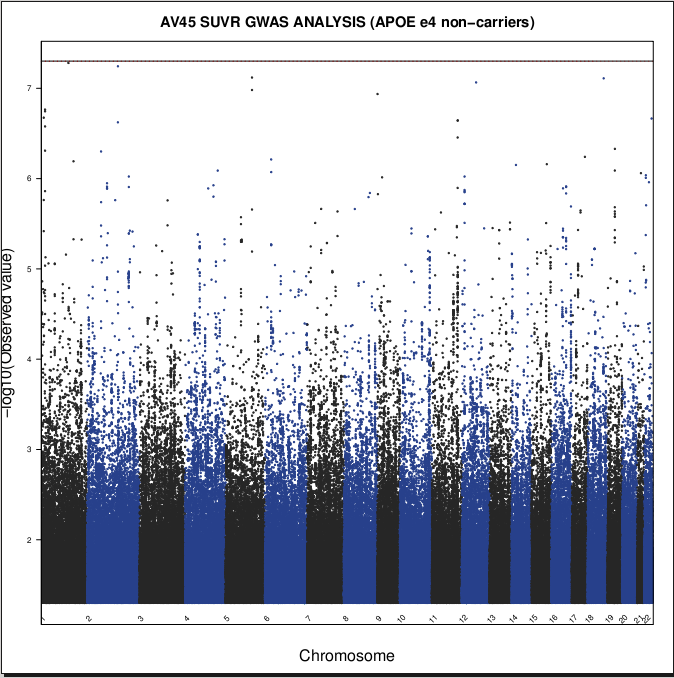


(5)


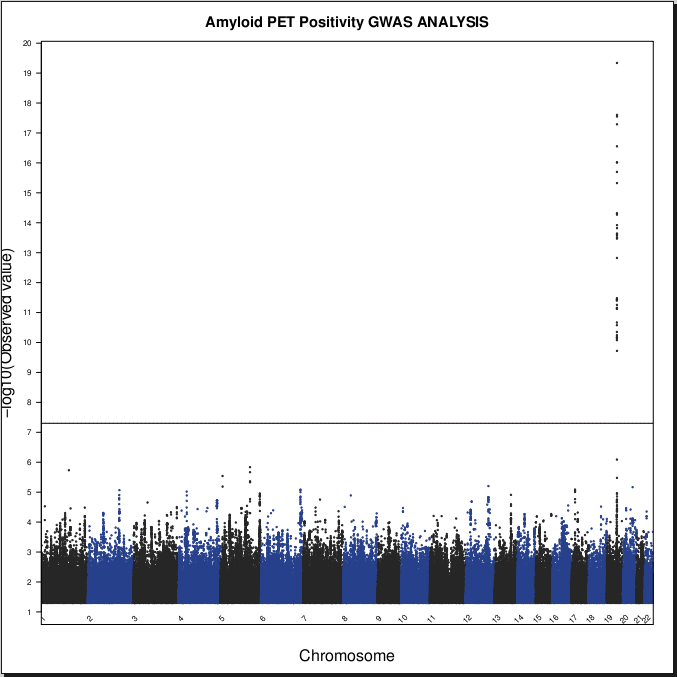


(6)


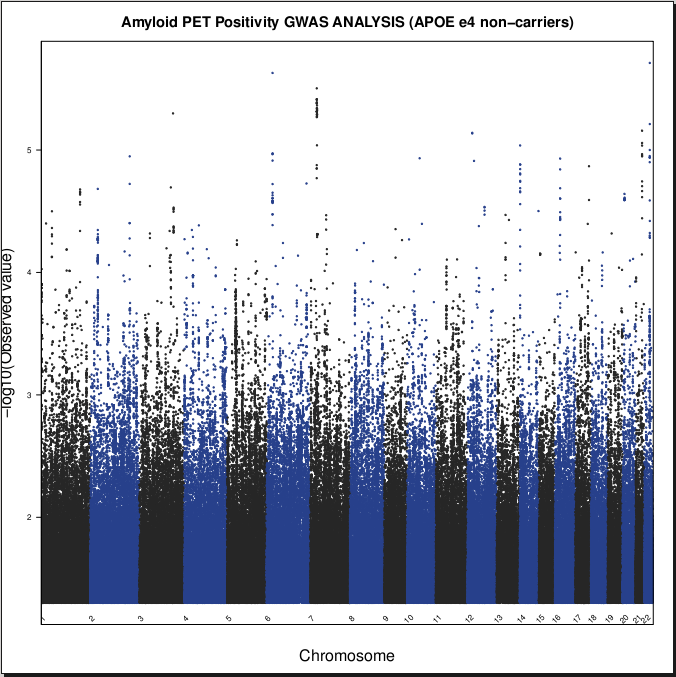


**Figure D: Manhattan plots of rate of cognitive decline GWAS in LMCI.** (1) European ancestry cohort (with population outlier removal); (2) cohort of all races (without population outlier removal). The dotted line indicates genome wide significance threshold of 5x10^-8^. Only variants with p < 0.05 are shown.

(1)

**
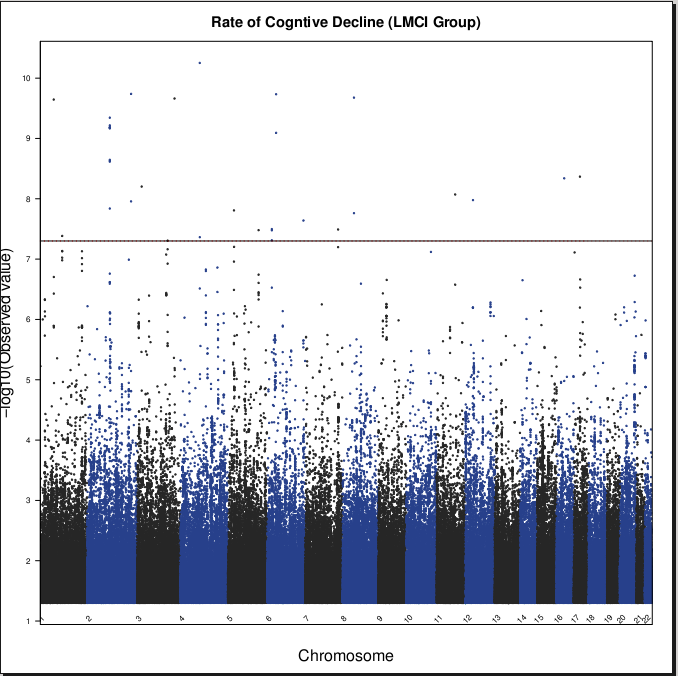
**

(2)


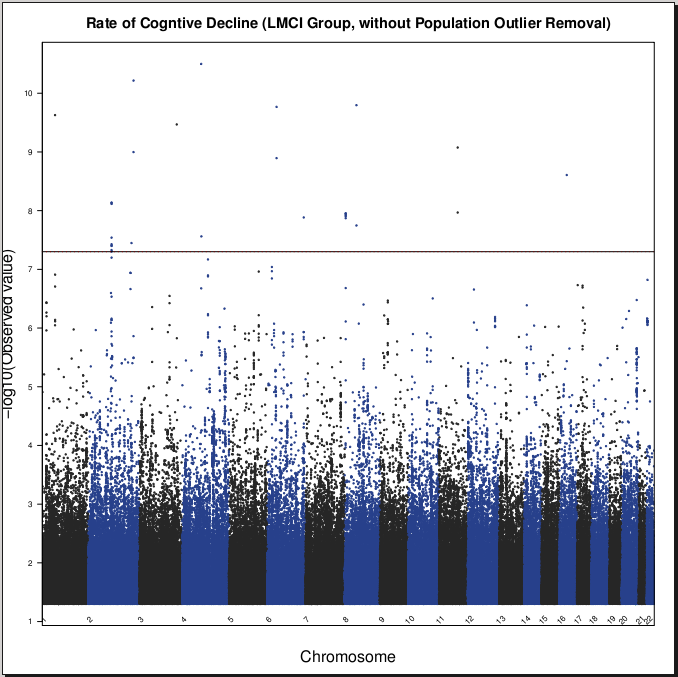


**Figure E: The impact of rs10509663-G dosage on disease progression as measured by CDR-SB in LMCI population** (1) mean, (2) least-squared mean

(1)


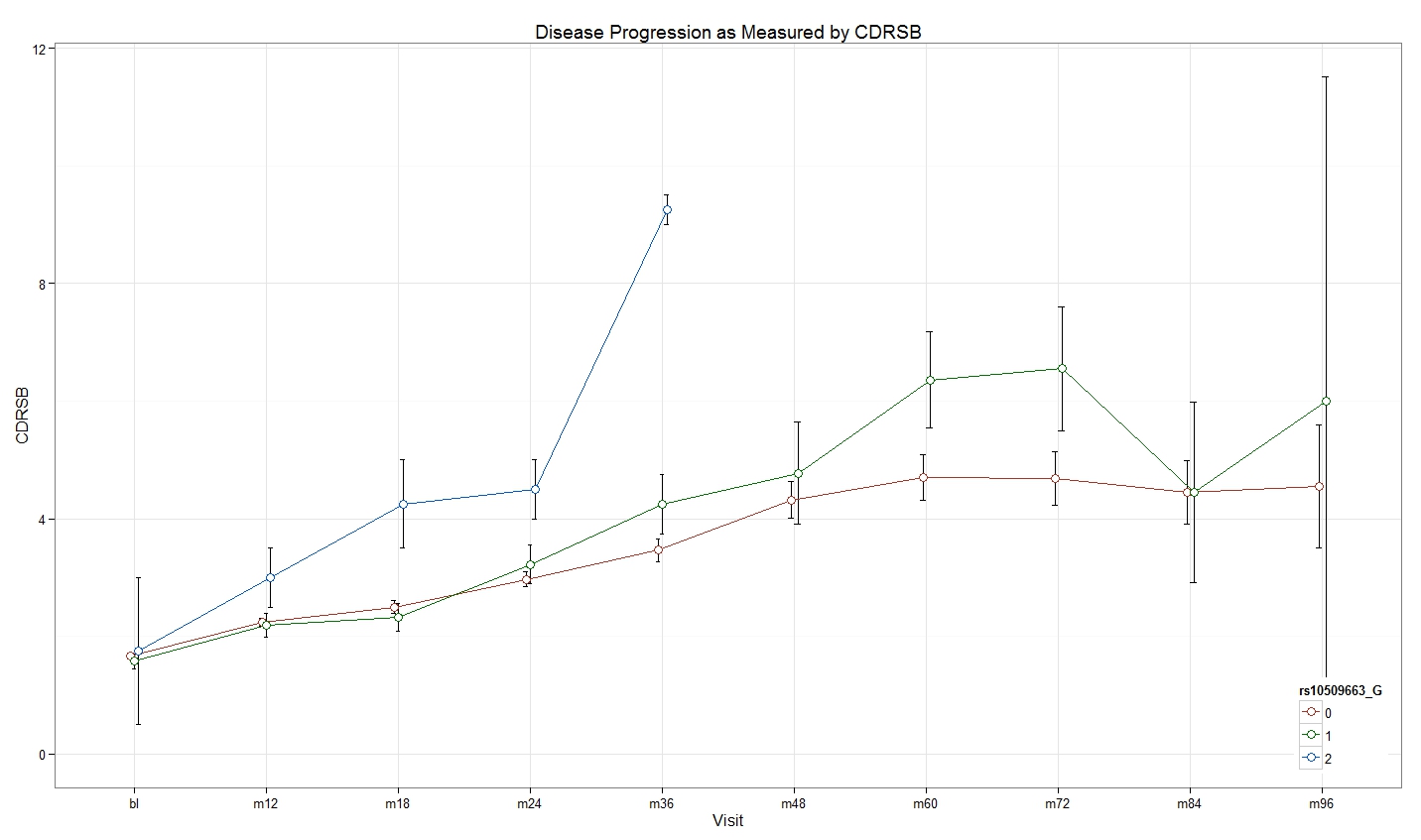


(2)

**Table A: Description of the analysis cohorts.** The genomic inflation factor (GIF) for each GWAS is included in the last two columns. All GWAS have GIF < 1.03 after population outlier removal and principal component correction. The GIF was reported based on the directly genotyped markers.

| **End point** | **Sample Size for the last column (n)** | **Genotyping Platform** | **GIF^a^ with covariates excluding PCs before population outlier removal** | **GIF^a^ with covariates including PCs after population outlier removal** |
| --- | --- | --- | --- | --- |
| Aβ_1-42_ | 340 | Illumina Human610-Quad BeadChip | 1.00438 | 1 |
| Aβ_1-42_ | 344 | Illumina Omni2.5 BeadChip | 1.02899 | 1 |
| Aβ_1-42_ | 172 | Illumina OmniExpress BeadChip | 1 | 1 |
| t-Tau | 335 | Illumina Human610-Quad BeadChip | 1.00414 | 1.0046 |
| t-Tau | 338 | Illumina Omni2.5 BeadChip | 1.13958 | 1 |
| t-Tau | 157 | Illumina OmniExpress BeadChip | 1 | 1 |
| t-Tau:Aβ_1-42_ ratio | 335 | Illumina Human610-Quad BeadChip | 1.01264 | 1.00893 |
| t-Tau:Aβ_1-42_ ratio | 338 | Illumina Omni2.5 BeadChip | 1.15237 | 1 |
| t-Tau:Aβ_1-42_ ratio | 157 | Illumina OmniExpress BeadChip | 1 | 1 |
| p-Tau_181P_ | 341 | Illumina Human610-Quad BeadChip | 1 | 1 |
| p-Tau_181P_ | 343 | Illumina Omni2.5 BeadChip | 1.06925 | 1 |
| p-Tau_181P_ | 171 | Illumina OmniExpress BeadChip | 1.02288 | 1.0011 |
| p-Tau_181P_:Aβ_1-42_ ratio | 340 | Illumina Human610-Quad BeadChip | 1.00538 | 1.0049 |
| p-Tau_181P_:Aβ_1-42_ ratio | 343 | Illumina Omni2.5 BeadChip | 1.0877 | 1 |
| p-Tau_181P_:Aβ_1-42_ ratio | 171 | Illumina OmniExpress BeadChip | 1.00886 | 1 |
| florbetapir PET (not correcting for APOE ε4) | 633 | Illumina Omni2.5 BeadChip | 1.00394 | 1 |
| florbetapir PET (not correcting for APOE ε4) | 250 | Illumina OmniExpress | 1.00009 | 1 |
| florbetapir PET (correcting for APOE ε4) | 627 | Illumina Omni2.5 BeadChip | 1 | 1 |
| florbetapir PET (correcting for APOE ε4) | 247 | Illumina OmniExpress | 1 | 1 |
| florbetapir PET (ε4 carriers) | 263 | Illumina Omni2.5 BeadChip | 1 | 1 |
| florbetapir PET (ε4 non-carriers) | 370 | Illumina Omni2.5 BeadChip | 1.0082 | 1.0072 |
| Amyloid PET positivity | 350 cases, 296 controls | Illumina Omni2.5 BeadChip | 1 | 1.00477 |
| Amyloid PET positivity (ε4 non-carriers) | 147 cases, 232 controls | Illumina Omni2.5 BeadChip | 1.01767 | 1.01536 |
| Rate of cognitive decline (ΔCDRSB/duration) in LMCI subgroup^b^ | 519 | Overlapping variants between Illumina Human610-Quad, OmniExpress, and Omni2.5 BeadChips | 1 | 1 |
| Rate of cognitive decline (ΔCDRSB/duration) in LMCI subgroup | 472 | Overlapping variants between Illumina Human610-Quad, OmniExpress, and Omni2.5 BeadChips | 1 | 1 |

^a^ Genomic inflation factor based on directly genotyped markers

^b^ Without population outlier removal (a.k.a. all races)

**Table B: Basic demographic characteristics of the two ADNI florbetapir PET sample sets**

|  | Omni2.5  (N=661) | OmniExpress  (N=291) |
| --- | --- | --- |
| Sex, n (%) |  |  |
| F | 298 (45.1) | 138 (47.4) |
| M | 363 (54.9) | 153 (52.6) |
| Age at baseline, years |  |  |
| Mean (SD) | 73.2 (7.0) | 72.2 (7.3) |
| Median (Range) | 73.1 (55.0, 91.4) | 72.5 (48.1, 89.3) |
| Baseline Clinical Diagnosis, n (%) |  |  |
| CN | 220 (33.3) | 19 (6.5) |
| SMC | N/A | 64 (2.2) |
| EMCI | 214 (32.3) | 67 (23.0) |
| LMCI | 182 (27.5) | 61 (21.0) |
| AD | 45 (6.8) | 80 (27.5) |
| APOE ε4, copy, n (%)^a^ |  |  |
| 0 | 387 (59.1) | 81 (42.0) |
| 1 | 222 (33.9) | 82 (42.5) |
| 2 | 46 (7.0) | 30 (15.5) |
| Missing Data | 6 | 98 |
| CDR-SB 0 n (%) | 207 (31.3) | 76 (26.1) |
| Peak florbetapir SUVR |  |  |
| Mean (SD) | 1.21 (0.23) | 1.24 (0.23) |
| Median (Range) | 1.13 (0.86, 2.06) | 1.21 (0.83, 1.93) |

^a^ APOE ε4 data in this table is from adnimerge table. Due to the substantial missing APOE ε4 data, we use best guess imputed e4 copy number in the genetic association analysis

**Table C: Basic demographic characteristics of the ADNI rate of cognitive decline sample set**

|  | LMCI  (N=540) |
| --- | --- |
| Sex, n (%) |  |
| F | 209 (38.7) |
| M | 331 (61.3) |
| Age at baseline, years |  |
| Mean (SD) | 73.9 (7.6) |
| Median (Range) | 74.3 (48.1, 91.4) |
| Genotyping platform, n (%) |  |
| Human610-Quad | 224 (41.5) |
| Omni2.5 | 248 (45.9) |
| OmniExpress | 68 (12.6) |
| APOE ε4, copy, n (%)^a^ |  |
| 0 | 245 (46) |
| 1 | 223 (42) |
| 2 | 69 (13) |
| Missing Data | 3 |
| Baseline CDR-SB |  |
| Mean (SD) | 1.65 (0.93) |
| Median (Range) | 1.5 (0.5, 5.5) |
| Baseline MMSE |  |
| Mean (SD) | 27.2 (0.23) |
| Median (Range) | 27 (23, 30) |
| Rate of cognitive decline |  |
| Mean (SD) | 0.76 (1.44) |
| Median (Range) | 0.5 (-3.0, 16) |
| Missing Data | 17 |

**Reference**

1. Purcell, S. *et al.* PLINK: a tool set for whole-genome association and population-based linkage analyses. *Am J Hum Genet* **81**, 559-75 (2007).

2. Price, A.L. *et al.* Principal components analysis corrects for stratification in genome-wide association studies. *Nat Genet* **38**, 904-9 (2006).

3. Patterson, N., Price, A.L. & Reich, D. Population structure and eigenanalysis. *PLoS Genet* **2**, e190 (2006).

4. Marchini, J., Howie, B., Myers, S., McVean, G. & Donnelly, P. A new multipoint method for genome-wide association studies by imputation of genotypes. *Nat Genet* **39**, 906-13 (2007).

5. Howie, B.N., Donnelly, P. & Marchini, J. A flexible and accurate genotype imputation method for the next generation of genome-wide association studies. *PLoS Genet* **5**, e1000529 (2009).

6. Marchini, J. & Howie, B. Genotype imputation for genome-wide association studies. *Nat Rev Genet* **11**, 499-511 (2010).

7. Howie, B., Marchini, J. & Stephens, M. Genotype imputation with thousands of genomes. *G3 (Bethesda)* **1**, 457-70 (2011).

8. Howie, B., Fuchsberger, C., Stephens, M., Marchini, J. & Abecasis, G.R. Fast and accurate genotype imputation in genome-wide association studies through pre-phasing. *Nat Genet* **44**, 955-9 (2012).

9. https://mathgen.stats.ox.ac.uk/impute/ALL_1000G_phase1integrated_SHAPEIT2_impute.nosingleton.tgz.

10. Zhao, J.H. gap: genetic analysis package. *Journal of Statistical Software* **23**, 1-18 (2007).

11. Pruim, R.J. *et al.* LocusZoom: regional visualization of genome-wide association scan results. *Bioinformatics* **26**, 2336-7 (2010).

12. Lambert, J.C. *et al.* Meta-analysis of 74,046 individuals identifies 11 new susceptibility loci for Alzheimer's disease. *Nat Genet* **45**, 1452-8 (2013).

13. Cruchaga, C. *et al.* GWAS of cerebrospinal fluid tau levels identifies risk variants for Alzheimer's disease. *Neuron* **78**, 256-68 (2013).

14. Ramanan, V.K. *et al.* APOE and BCHE as modulators of cerebral amyloid deposition: a florbetapir PET genome-wide association study. *Mol Psychiatry* **19**, 351-7 (2014).

15. Hu, X. *et al.* Genome-wide association study identifies multiple novel loci associated with disease progression in subjects with mild cognitive impairment. *Transl Psychiatry* **1**, e54 (2011).
